# Insight into the Characterization of Two Female Suppressor Gene Families: *SOFF* and *SyGI* in Plants

**DOI:** 10.3390/genes16030280

**Published:** 2025-02-26

**Authors:** Yanrui Zhu, Zeeshan Ahmad, Youjun Lv, Yongshan Zhang, Guodong Chen

**Affiliations:** 1 Department of Agronomy, College of Agriculture, Tarim University, Alar 843300, China; 19899079832@163.com; 2 Key Laboratory of Genetic Improvement and Efficient Production for Specialty Crops in Arid Southern Xinjiang of Xinjiang Corps, Tarim University, Alar 843300, China; 3 National Key Laboratory of Cotton Bio-Breeding and Integrated Utilization, Institute of Cotton Research, Chinese Academy of Agricultural Sciences, Anyang 455000, China; zeeshanpbg@aup.edu.pk; 4 Anyang Institute of Technology, Anyang 455000, China; 20160404@ayit.edu.cn

**Keywords:** dioecism, Suppressor of Female Function (*SOFF*), Shy Girl (*SyGI*), *Asparagus*, kiwi fruit, *Arabidopsis thaliana*, genome-wide studies (GWSs), sex determination

## Abstract

Background/Objectives: The Suppressor of Female Function (*SOFF*) and Shy Girl (*SyGI*) gene families play vital roles in sex determination in dioecious plants. However, their evolutionary dynamics and functional characteristics remain largely unexplored. Methods: Through this study, a systematic bioinformatics analysis of *SOFF* and *SyGI* families was performed in plants to explore their evolutionary relationships, gene structures, motif synteny and functional predictions. Results: Phylogenetic analysis showed that the *SOFF* family expanded over time and was divided into two subfamilies and seven groups, while *SyGI* was a smaller family made of compact molecules with three groups. Synteny analysis revealed that 125 duplicated gene pairs were identified in Kiwifruit where WGD/segmental duplication played a major role in duplicating these events. Structural analysis predicted that *SOFF* genes have a DUF 247 domain with a transmembrane region, while *SyGI* sequences have an REC-like conserved domain, with a “barrel-shaped” structure consisting of five α-helices and five β-strands. Promoter region analysis highlighted their probable regulatory roles in plant development, hormone signaling and stress responses. Protein interaction analysis exhibited only four *SOFF* genes with a close interaction with other genes, while *SyGI* genes had extensive interactions, particularly with cytokinin signal transduction pathways. Conclusions: The current study offers a crucial understanding of the molecular evolution and functional characteristics of *SOFF* and *SyGI* gene families, providing a foundation for future functional validation and genetic studies on developmental regulation and sex determination in dioecious plants. Also, this research enhances our insight into plant reproductive biology and offers possible targets for breeding and genetic engineering approaches.

## 1. Introduction

Sex is an important character of plants and is the key feature to plant adaptation to environmental changes and species reproduction. In the long process of plant evolution, sex differentiation occurs constantly and produces male and female organs [1]. Nearly 90% of the angiosperm species produce bisexual flowers having both pistils and stamens, while the remaining 10% produce unisexual flowers with either pistils or stamens, which are called female or male flowers, respectively [2]. In monoecious plants, both female and male flowers coexist on the same plant, while females produce only female flowers and males have only male flowers in dioecious species [3,4]. Asparagus (*Asparagus officinalis* L.) and Kiwifruit (*Actinidia chinensis* L.) are two important and typical dioecious plants. Asparagus, one of the most important vegetables in the world, is a perennial plant species subordinating to the genus *Asparagus*, in which most of the plants are dioecious. Most species in the genus *Actinidia*, including the kiwifruit, are also dioecious, and are considered one of the most important fruits around the world [5,6].

There are two genetic theoretical models of dioecious evolution, the “One Gene” model and the “Two Genes” model. The “Two Genes” model proposed by Charlesworth et al. in 1978 has been more widely accepted [7]. According to the “Two Genes” model, dioecious traits evolved from hermaphroditic plants through female bisexual flower intermediates in a potential sequence of generating a recessive male sterility mutation first followed by a dominant female sterility mutation. These mutations most likely occur in two separate genes that are involved in sex determination in dioecious plants and are closely linked to an inhibited recombination of sex-determining regions (SDRs) on sex chromosomes [8,9]. Genetically, both asparagus and kiwifruit are dioecious species with the XY sex-determination system and have a pair of isotypic sex chromosomes. The SDR on the Y chromosome contains both a male activator gene and a female suppressor gene, while the corresponding region of the X chromosome lost key genes related to male function, resulting in the male plant with the XY chromosome and the female plant with the XX chromosome [10]. The “Two Genes” model is supported by recent advances in experimental studies on sex determination in kiwifruit and asparagus [5,11,12,13,14,15,16].

Two genes in the SDR on the Y chromosome, *AspSOFF* (Suppressor of Female Function) and *AspTDF1* (Defective in Tapetum Development and Function 1), are associated with sex determination in asparagus, among which *SOFF* is a female suppressor gene. Male asparagus plants with *SOFF* gene knockout can produce monoecious individuals, while the MYB transcription factor *AspTDF1* gene is a male activator, which is only expressed in males, and is necessary for male organ (anther) development [13,15,16]. Kiwifruit sex is also determined by two genes located in the SDR of the Y chromosome. Among them, the C-type *RR* (Cytokinin-Responsive Regulator) gene, known as *SyGI* (Shy Girl), acts as a female suppressor (*SuF*) by inhibiting carpel development [5,12], while the male promoter gene *FrBy* (Friendly Boy) determines the development of male characteristics in kiwifruit [12]. The *FrBy* gene is associated with *MTR1* (Microspore and Tapetum Regulator 1) genes in rice. *MTR1* gene promotes tapetum degradation in rice, leading to male infertility [17], and the introduction of *FrBy* in female kiwifruit can produce hermaphroditic individuals [12].

Recently, a new unified genetic network model for sex determination in monoecy and dioecy plants has been proposed, including female suppressors (*SOFF* in asparagus and *SyGI* in kiwifruit), female and male promoters (*TDF1* in asparagus and *FyBy* in kiwifruit) and high-level sex switches [5,12,16,18]. According to this genetic model, the female suppressors that inhibit pistil development, namely asparagus *SOFF* and kiwi fruit *SyGI*, are integrated into this unified molecular genetic model that controls sex development [18]. Although, all these previous findings have used physical and molecular experiments to identify the functional roles of genes, a crucial knowledge gap still exists regarding their evolutionary history, genetic variations and larger regulatory networks across species. The degree to which these genes exhibit conserved or divergent mechanisms across many diecious systems is currently unidentified, and little is known about how they might interact with sex determining factors. Also, the structural differences, cis-regulatory components and transcriptional dynamics that affect their expression in various developmental stages and environments are yet to be investigated.

To fill these knowledge gaps, we have conducted a systematic bioinformatics analysis of the *SOFF* and *SyGI* gene families, using comparative genomics, phylogenetics, sequence motif analysis and transcriptome profiling to uncover their evolutionary relationships and regulatory mechanisms. This study will enhance our understanding of the genetic basis of sex determination and deliver evidence for the comprehensive interpretation of the evolution of dioecy in plants. This study will lay the foundation for future experimental validations to study the functions of *SOFF* and *SyGI*, as well as the molecular mechanisms and breeding applications in dioecious crop species.

## 2. Materials and Methods

### 2.1. Identification of SOFF and SyGI Family Genes

The full-length protein sequences of *SOFF* and *SyGI* were used as queries and the genomic as well as protein sequences of different species were procured from phytozome (https://phytozome.jgi.doe.gov/pz/portal.html/, accessed on 20 August 2024) and relevant databases, including *Physcomitrella patens* (v.3.3) [19], *Sphagnum fallax* (v1.0), *Selaginella moellendorffii* (v.1.0) [20], *Amborella trichopoda* v.1.0 [21], *Oryza sativa* subsp. japonica (v.7.0) [22], *A. officinal* (v.1.1) [15], *Asparagus setaceus* (v.1.0) [23], *Aquilegia coerulea* v.3.1 [24], *A. chinensis* (v.3.0) [25], *Gossypium raimondii* (v.5.0) [26] and *A. thaliana* (v1.1) [27] as the target. BlastP was also used with the Tbtool software (v1.082) [28]. These species represent key evolutionary lineages, including bryophytes, ferns, monocots, and eudicots. The other physiochemical properties were also noted from the same databases, and some were measured manually by downloading their genomic sequence files and setting all the parameters as follows: E-value ≤ 1 × 10^−5^, sequence alignment length > 50% of the length of asparagus *SOFF* (*evm. model. AsparagusV1_01. 231.V1.1*, 451 aa) or Kiwifruit *SyGI* (*Actinidia33667.1*, 148aa), sequence length of target sequence of *SyGI* gene < 300 aa.

### 2.2. Phylogenetic Analysis of SOFF and SyGI Gene Families

Sequence alignment and phylogenetic analysis were performed using MEGA v.10.2 software for the *SOFF* and *SyGI* protein sequences [29]. The neighbor-joining (NJ) method was used to construct NJ trees. The Jones–Taylor–Thornton + Gamma distributed (JTT + G) matrix model with the Gamma parameter set to default as 5, and the comparison sites excluded with coverage lower than 60% were used to construct the phylogenetic tree. The ML phylogenetic analysis output was submitted to the iTOL website (https://itol.embl.de/ accessed on 2 September 2024) for online visualization [30].

### 2.3. Evolutionary Conserved Module (ECM) Analysis of the SOFF and SyGIs

Evolutionary history was analyzed online by CLIME v.1.0 software [31]. CLIME software can divide a group of genes into several Evolutionarily Conserved Modules and further identify and screen out other genes based on their evolutionary (species distribution) characteristics in the biological world. Genes from the same module have similar evolutionary (species distribution) characteristics in the biological world. In this analysis, the *SOFF* and *SyGI* orthologous genes identified from the *A. thaliana* genome were submitted to CLIME for online analysis (https://www.gene-clime.org/, accessed on 15 September 2024) to categorize them into various groups (ECMs).

### 2.4. Collinearity and Selective Pressure Analysis

To generate the collinearity blocks throughout the entire genome, the protein sequences were analyzed by MCSCAN in Tbtool software v1.082 [32]. The collinearity pairs from *SOFF* and *SyGI* gene families were extracted to draw the collinearity maps within the gene families using CIRCOS and Tbtools v1.082. The MEGA v.10.2 program was used to align the duplicated gene pairs, and TBtool software was used to compute the synonymous (Ks) and non-synonymous (Ka) substitution rates. According to the Ka/Ks ratio, a ratio less than one denotes negative or purifying selection, a ratio greater than one denotes positive selection, and a ratio equal to one denotes neutral selection. Lastly, each duplicated *SOFF* and *SyGI* gene pair’s selection pressure was calculated.

The *SOFF* and *SyGI* gene sequences were submitted to the SMART (a Simple Modular Architecture Research Tool) for the identification and annotation of protein domains and the analysis of protein domain architectures (http://smart.embl-heidelberg.de/, accessed 1 October 2024), with all the parameters set to default on the website [33]. All *SOFF* protein sequences were then submitted to TMHMM v.2.0 (https://services.healthtech.dtu.dk/service.php?TMHMM-2.0, accessed on 8 October 2024) for the prediction of transmembrane helices [34]. The conserved protein sequences were analyzed online on the MEME suite v5.5.5 (Multiple Em for Motif Elicitation) website (https://meme-suite.org/meme/tools/meme, accessed on 17 October 2024), and the module number was set to 10, with other parameters set to the default setting [35,36]. The figures of domain architectures and MEME motifs were drawn by TBtool v1.082 software [28].

### 2.5. Prediction of Tertiary Structure and Active Site of SOFF and SyGI Genes

Online predictions of protein 3D structure and ligand binding sites, including the homolog protein of *SOFF* in *O. sativa Os11G33394.1* and the homolog of *SyGI At3g04280.1* in *A. thaliana*, were performed by AlphaFold [37] and P2Rank [38]. Subsequently, the 3D structure PDB file was downloaded and was submitted to the PrankWeb website (https://prankweb.cz/, accessed on 25 October 2024) to further predict ligand binding sites by using the “Custom Structure” method. The tertiary structures of asparagus *SOFF* protein *Aspofficinalis01.231* and Kiwifruit *SyGI* protein *Actinidia33667* were predicted on the Swiss-Model website (https://swissmodel.expasy.org/interactive, accessed on 6 November 2024) using *Os11G33394* protein and *At3G04280.1* protein as templates, respectively.

### 2.6. Promoro Region Analysis of SOFFs and SyGIs in A. thaliana

The cis-acting elements were predicted by analyzing the promoter regions of *A. thaliana* for the *SOFF* and *SYGI* genes, and any differences between them were compared. For both gene families, the DNA sequences of the 2000 bp upstream region of the start codon were obtained from the Phytozome v.13 website (https://phytozome-next.jgi.doe.gov/, accessed on 19 November 2024) for chosen species. The sequences were then uploaded to the PlantCARE website (https://bioinformatics.psb.ugent.be/webtools/plantcare/html/ accessed on 25 November 2024) to forecast and examine the *cis*-regulator elements in the promoter region of both families that are linked to phytohormones, plant growth and development, stressors, and light responses. The *cis*-acting components’ visuals were created and visualized using the TBtool (v1.082) software.

### 2.7. STRING Analysis of Protein–Protein Interaction Network Functional Enrichment of SOFFs and SyGIs in A. thaliana

Analysis of protein–protein interaction of 19 *SOFFs* and 2 *SyGIs* in *Arabidopsis* was performed on the STRING website (https://cn.string-db.org/, accessed on 4 December 2024). The full network containing both functional and physical protein associations was analyzed based on the STRING database, which is built on published sources of experiments, databases, and co-expression profiles. The minimum required interaction score was set as 0.700, and the maximum numbers of displayed interactors from the first and second round of search were set to be no higher than 20. Other parameters were set to default.

## 3. Results

### 3.1. Identification and Phylogenetic Analysis of SOFF Gene Family

The results of gene identification revealed a total of 192 *SOFF* family genes, where only 1 (1) homolog was identified in the genome of the ancestral plant species *S. fallax*, 0 for both *P. patens* and *S. moellendorffii*, and 12 for *A. trichopoda*. Also, many *SOFF* homologous genes were found in the genomes of some other plant species, such as *A. coerulea* (30), *G. raimondii* (28), *A. chinensis* (42) and *A. thaliana* (19). Meanwhile, many *SOFF* homologs were detected in the monocots, including *O. sativa* and in *A. setaceus*, with each having 26 and 8 in *A. officinal* (Table S1a). These results indicated that the *SOFF* gene family had an obvious expansion during evolution, with an increasing number of genes of the *SOFF* gene family throughout the plant kingdom. Meanwhile, many tandem replication events in the entire evolutionary expansion process of the *SOFF* gene family have been recognized, such as *AmTr16.321*, *AmTr16.322*, *AmTr16.323*, *AmTr16.326*, *AmTr16.327*, *AmTr16.329* in the genome of the angiosperm ancestor *A. trichopoda*, and *AT3G50120*, *AT3G50130*, *AT3G50140*, *AT3G50150*, *AT3G50160*, *AT3G50170*, *AT3G50180*, *AT3G50190* in *A. thaliana*, *Aqcoe5G364900*, *Aqcoe5G365000*, *Aqcoe5G365100*, *Aqcoe5G365700* in *A. coerulea*, etc.

**Figure 1:**
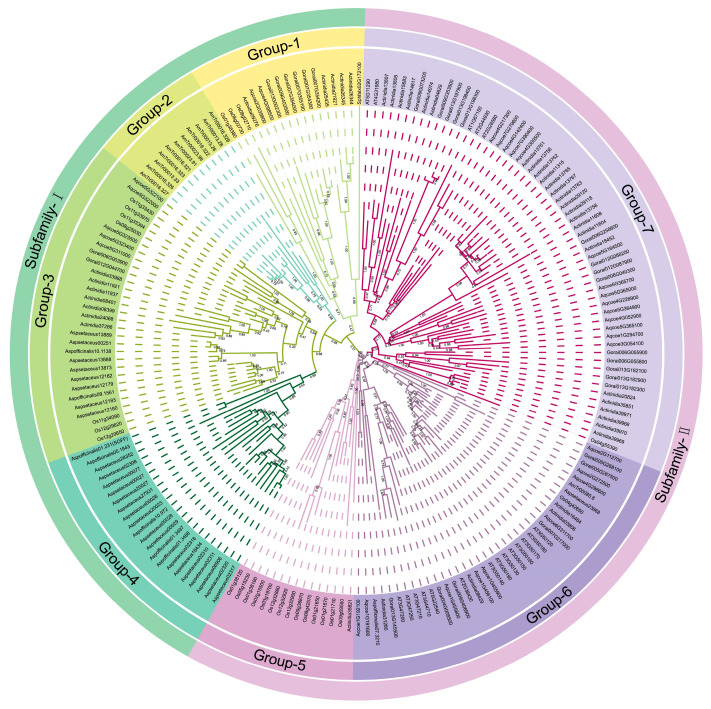
The Maximum Likelihood (ML) phylogenetic tree of *SOFF* family. The Maximum Likelihood (ML) phylogeny trees were constructed using MEGA 10.2 software. Bootstrap values above 0.5 from 1000 replicates are shown at each node. Different colors represent various phylogenetic groups.

The *SOFF* gene family can be divided into two subfamilies (Subfamily I and Subfamily II) and seven groups according to phylogenetic analysis (Figure 1). Subfamily I contains four groups (Group 1–4). In Group 1, only one *SOFF* homolog gene from *S. fallax* is present, indicating that the *SOFF* gene might first appear in bryophytes, while the 11 *SOFF* genes listed in Group 2 are all from the ancestor of the angiosperm *A. trichopoda*. The significant increase in the *SOFF* genes compared with *S. fallax* indicates that the SOFF family has expanded significantly in the ancestral species of angiosperms, and many tandem duplications of *SOFF* genes in *A. trichopoda* indicate that tandem duplication may be the main driving force of the *SOFF* gene family expansion. Group 3 constituted 31 *SOFF* homologs from dicotyledonous and monocotyledonous plants, while Group 4 contained 22 *SOFF* genes, and all of them were derived from *A. officinal* and *A. setaceus*. Subfamily II consisted of three groups including Group 5 to 7. Group 5 was mainly composed of *SOFF* homolog genes from monocotyledon *O. sativa* and one from dicotyledon *A. chinensis*. Group 6 was composed of *SOFF* genes from dicotyledon plants such as *A. coerulea*, *G. raimondii*, *A. chinensis*, and *A. thaliana*, as well as one *SOFF* gene from each of *A. trichopoda*, *O. sativa* and *A. officinal*. In Group 7, the majority of the *SOFF* homologs were occupied by dicotyledons, including only one *SOFF* homolog from *O. sativa*.

### 3.2. Identification and Phylogenetic Analysis of SyGI Gene Family

Using the *SyGI* sequences of Kiwifruit as the query, a total of 35 *SyGI* gene sequences were obtained. The results showed that the number of *SyGI* genes did not expand significantly with the WGD (Whole Genome Duplications) in the evolutionary history of plants, except for one *SyGI* gene that had been identified in the genomes of the more ancient moss species *P. patens* and *S. fallax*, respectively (Table S1b).

**Figure 2:**
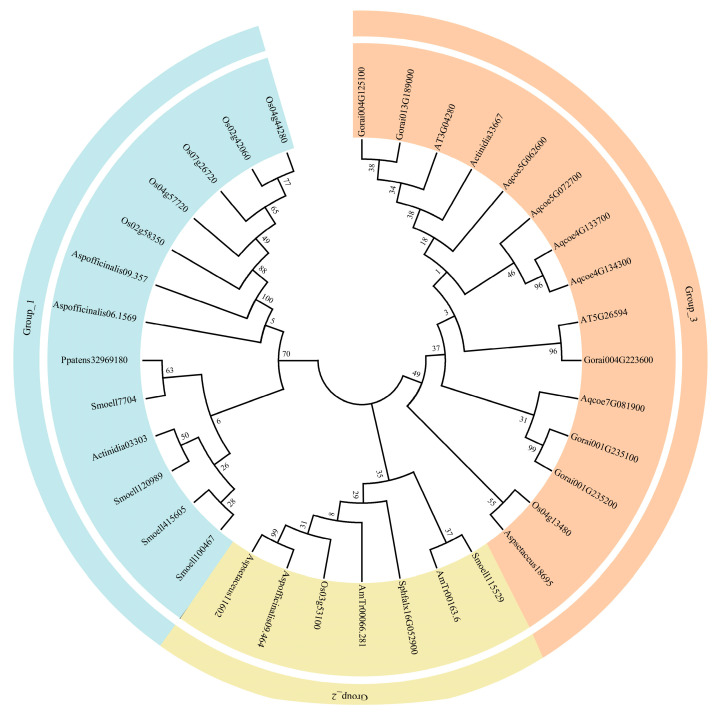
The Maximum Likelihood (ML) phylogeny trees of *SyGI* family genes. The Maximum Likelihood (ML) phylogeny trees were constructed using MEGA 10.2 software. Bootstrap values above 0.5 from 1000 replicates are shown at each node. Different colors represent various phylogenetic groups.

Phylogenetic analysis showed that the *SyGI* gene family can be divided into three groups: Group 1, 2, and 3 (Figure 2). The genes in Group 1 and 2 were derived from the genomes of ancient mosses, such as *P. patens* and *S. fallax*, and of the ancestors of seed and angiosperm plants, such as *S. moellendorffii*, *A. trichopoda*, as well as from monocotyledons and dicotyledons such as *O. sativa*, *A. officinal* and *A. thaliana*. Unlike Groups 1 and 2, the genes in Group 3 were derived entirely from the monocotyledon and dicotyledon genomes, but none were derived from ancestral plant species.

### 3.3. Analysis of Evolutionary Conserved Characteristics of SOFF and SyGI Gene Family in Plants

**Figure 3:**
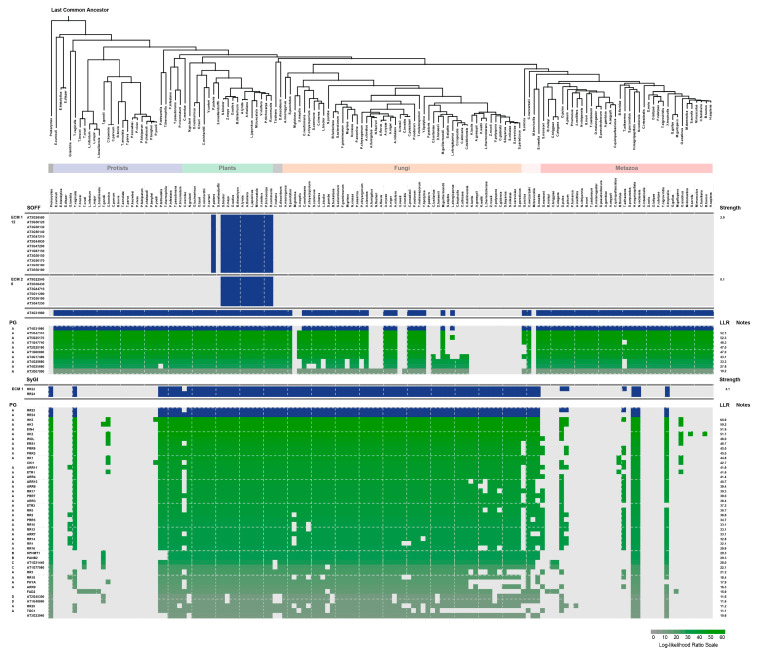
Overview of Evolutionarily Conserved Modules (ECMs) of *SOFF* and *SyGI* homologous genes in *A. thaliana*.

Nineteen (19) identified *SOFF* genes in *Arabidopsis* were submitted to CLIME analysis (Figure 3). The results showed that all 19 *SOFF* genes in *A. thaliana* could be divided into three ECMs according to the evolutionary characteristics of different genes in the biological world. Among them, 12 *SOFF* genes found in ECM 1 and 6 *SOFF*s in ECM 2 were only found in plant species like moss, but not in the protozoa, fungi, or animal kingdom. The difference between ECM1 and ECM2 is that all genes in the ECM1 module can be identified as orthologous genes in moss (*P. patens*), indicating that the origin of each gene in the ECM1 module can be traced back to moss, while no orthologs in ECM1 were detected in the ancestor of the seed plant *S. moellendorffii*, which might be caused by gene loss. The orthologs of the six ECM2 genes were derived from the common ancestor of monocotyledons and dicotyledons, but not in *P. patens* and *S. moellendorffii*, suggesting that the ECM2 module genes may be derived from the ancestors of angiosperms. Although there was only one *SOFF* gene (*AT4G31980.1*) in ECM3, its homology was widely present in the main lineages of protozoa, plants, fungi, and animals; however, it has been lost in some lineages of fungi and animals. Among the three evolutionary conservative modules, ECM1 and ECM2 modules failed to identify the existence of other genes with similar evolutionary historical characteristics (LLR ≥ 10). In addition, 21 genes with similar evolutionary history characteristics as the only *A. thaliana SOFF* gene *AT4G31980* in ECM3 were detected, among which 9 genes with highly similar evolutionary history characteristics (LLR ≥ 10, ECM_3+) were mainly PPPDE proteins with deubiquitinase functions (putative thiol peptidase family proteins) (Figure 3 and File S1).

Two *SyGI* homologous genes were identified in *Arabidopsis*, *ARR22* and *ARR24* that both belong to the *Arabidopsis* RR (Two-Component Response Regulator) family and belong to the same ECM due to the high similarity of evolutionary characteristics (Figure 3). *SyGI* genes have been widely identified in protists, plants, fungi, and animals, even in the most primitive prokaryotes. The results also showed that few *SyGI* genes were identified in lineages or species of protozoa and animals, suggesting that the homolog genes of *SyGI* were lost in these lineages or species, while *SyGI* genes are present in almost all plant and fungal species. Further ECM identification was carried out on *SyGI* genes, and 42 genes with similar evolutionary historical characteristics as *ARR22* and *ARR24* were identified (LLR ≥ 10, ECM_1+). These genes included 17 other *ARR* genes and 4 *APRR* (two-component Response Regulator like Pseudo-Response Regulator) genes belonging to the *ARR* gene family, but also having five genes of the HK (Histidine kinase) family (HK1-3, 5 and WOL), Ethylene Insensitive protein 4 (EIN4), Ethylene Receptor 1 (ETR1), Ethylene Response Sensor 1 (ERS1), etc. (Figure 3 and File S2).

### 3.4. Collinearity and Selective Pressure Analysis of SOFF and SyGI Genes

Synteny analysis was performed to identify the tandem duplication and whole genome duplication (WGD)/segmental duplications, and to clarify the mechanisms of *SOFF* and *SyGI* genes in *Asparagus officinials* and kiwi fruit, respectively. The syntenic analysis for *SOFF* genes in asparagus was not possible because of the only simple diploid genome of *Asparagus officinials* and most of the other species’ genomes of the asparagus family are yet to be fully sequenced. However, we compared the asparagus genome with five other genomes such as *Arabidopsis*, *Amborella tricopoda*, wheat, maize and tomato in various plant families separately to identify the comparative syntenic genes but did not find any syntenic and linked genes in these genomes for *SOFF* genes.

**Figure 4:**
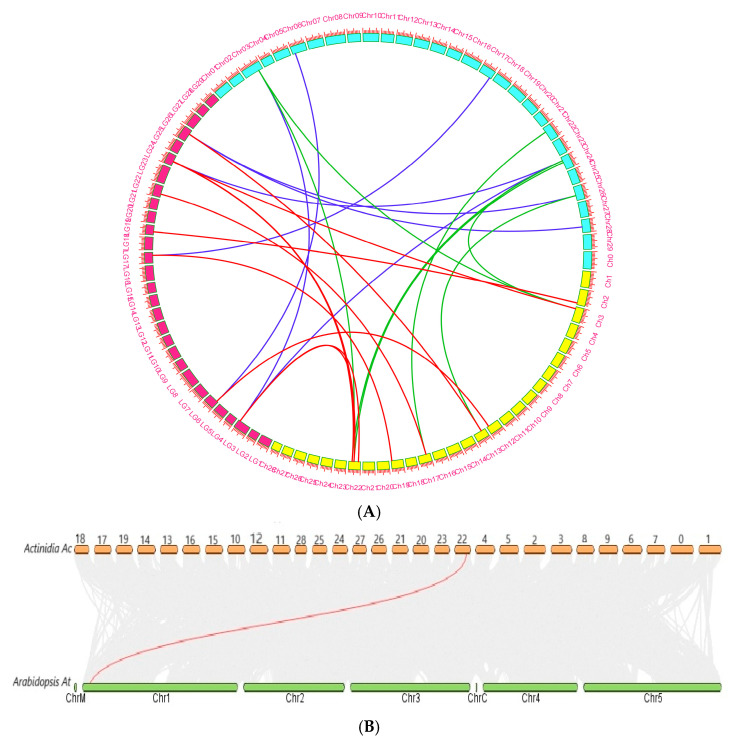
Collinearity and selective pressure analysis. (**A**) Collinearity analysis for *SyGI* genes in *Actinidia chenisis genomes.* Different colors represent various genomes, where yellow color indicates Hongyang3, red color represents Actinidia Red and sky-blue color represents Actinidia White genomes. (**B**) Dual synteny analysis of *Actinidia chenisis* with *A. thaliana*.

Synteny analyses of the *SyGI* genes were performed in three species of kiwi fruit by double comparison through multiple and pairwise alignment of *SyGI*s. The syntenic results revealed that *SyGI* genes are conserved among the three species of kiwifruit, and a total of 25 orthologous/paralogous gene pairs were identified including 11 gene pairs in Hong yang3, and 7 gene pairs for each Actinidia red and Actinidia white (Figure 4A). Interestingly, the identified gene pairs arose through segmental/whole genome duplication, which are indictive of evolutionary processes that have contributed to genome expansion and diversification in the species.

Based on the results, comparative syntenic maps of Kiwi fruit with two representative species, *Arabidopsis* and *Amborella tricopoda*, were constructed to further analyze the phylogenetic mechanisms of the *SyGI* gene family (Figure 4B). In the comparison between kiwi fruit and *Arabidopsis*, only a single gene pair showed a syntenic relationship, while with *Amborela tricopoda*, no relationship with any gene was identified.

Furthermore, to identify whether the Darwinian positive selection was correlated with the *SOFF* gene divergence post-duplication, the Ka/Ks values of 25 duplicated gene pairs were measured in three Kiwi fruit species in all combinations (Table S4). The results of Ka versus Ks revealed that ratios from 21 gene pairs were smaller than 1, suggesting that purifying selection pressure was the dominant force maintaining functional conservation with limited divergence after segmental/WGD. In addition, two other gene pairs *Actinidia37570.t1* and *DTZ79_22g03170* vs. *Actinidia03303.t1* displayed Ka/Ks ratios greater than 1, indicating that diversifying (positive) selection pressure is often linked to functional innovation and might undergo rapid evolution after duplication.

### 3.5. Primary Structure of SOFF and SyGI Genes

Most of the 192 *SOFF* genes have a transmembrane domain of around 20 amino acids at the C-terminal of the DUF247 domain and downstream (Figure S1). There were also some *SOFF* genes whose transmembrane domains were located upstream, inside, or downstream of the DUF247 domain, and very few SOFF proteins showed two transmembrane domains (Figure S1B). According to the primary structure of *SOFF* genes, the transmembrane domain with a total length of about 23 amino acid residues had 6 amino acid residues from the C-terminal of Motif5 and around 17 amino acid residues from the N-terminal of Motif6 (Figure S1D). It is worth noting that the Trp of the N-terminal of Motif6 was stringently conserved across all analyzed species. Downstream of the transmembrane domain is a very short intracellular sequence, suggesting that the main structure of *SOFF* genes was located on the non-cytoplasmic side of the cell membrane. Furthermore, a total of 15 motifs were identified where most of the sequences ranged from 5 to 15 motifs and Motifs 1,2 and 3 were conserved among all the gene sequences (Figure S1C).

**Figure 5:**
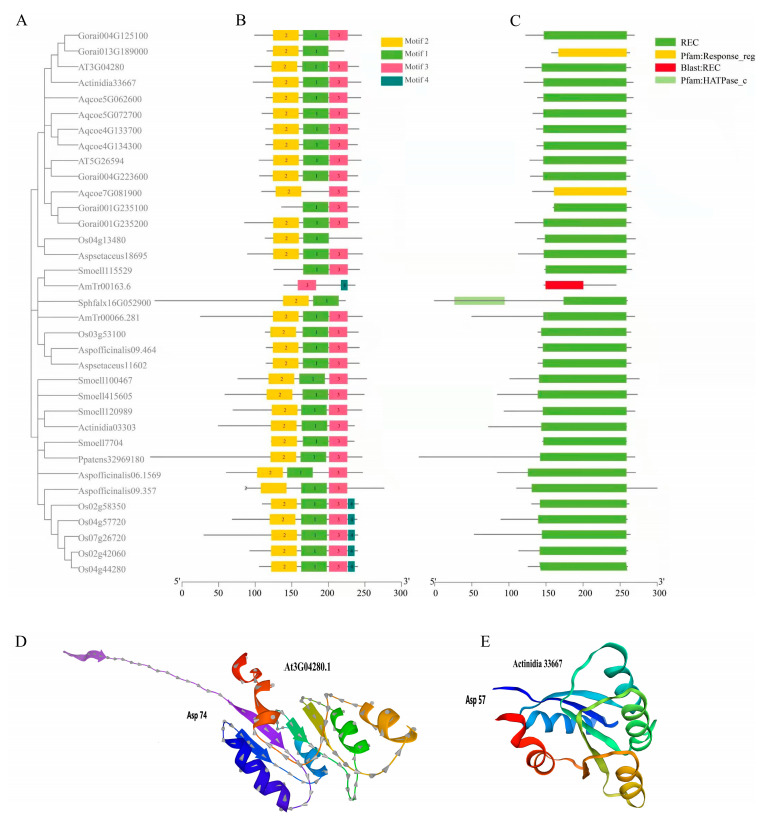
Schematic diagram of the motifs, domains and tertiary structure of *SyGI* proteins. (**A**) The NJ phylogenetic tree was constructed based on the full-length sequences of *SyGI* genes using MEGA 10.2 software. (**B**) The motif composition of *SyGI* proteins. (**C**) Schematic representation of the domain in *SyGI* genes. (**D**) The tertiary structure of *SyGI* homologous protein in *A. thaliana* (*At3G04280.1*). (**E**) Tertiary structure prediction of Kiwifruit *SyGI* genes (*Actinidia33667.1*) based on *AT3G04280.1* as a template. The *SyGI* domains are highlighted by red boxes and grey boxes, respectively. The length of protein can be estimated using the scale at the bottom.

The results of *SyGI* protein sequences detected four conserved domains and almost all of them were related to signal transduction, including the REC domain signatured for phosphoric group transfer (Figure 5). The REC-like domains, including the REC domain and its nearly identical REC domains, were characterized by the presence of a site that can be phosphorylated by histidine-kinase [39,40]. The REC-like domains exist primarily as a structural and functional module in the protein families involved in signal transduction, such as *HK* (Histidine kinase) family and *HP* (His-containing phosphotransfer proteins) family (Figure 5B) [41]. A total of 4 motifs were detected in 34 *SyGI* gene sequences, among which Motif 1, 2, and 3 existed in most of the *SyGI* proteins, while Motif 4 mainly existed in *SyGI* genes of rice. A few *SyGI* proteins only had two motifs (Figure 5B).

### 3.6. Tertiary Structural Prediction of SOFF and SyGI Homologous Proteins

**Figure 6:**
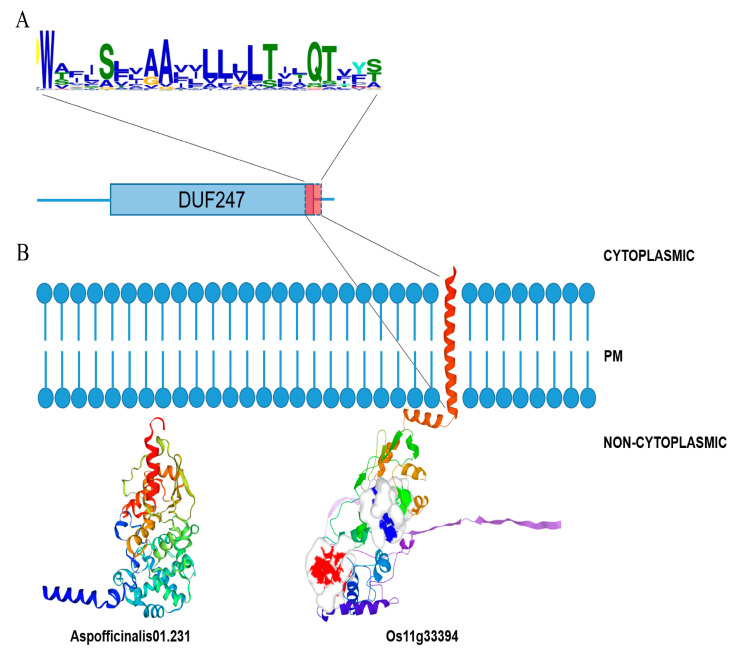
Schematic diagram of tertiary structure (including transmembrane domain) and subcellular localization of *SOFF* homologous proteins. (**A**) Sequence and primary structure of transmembrane domain of *SOFF* proteins. (**B**) Schematic diagram of transmembrane domain and subcellular localization of *SOFF* proteins.

*SOFF* family proteins are typically characterized by a conserved domain of DUF247 (Pfam03140), which is the only component of the CL03911 superfamily [32]. The structure prediction of the *SOFF* orthologous gene (*Os11G33394.1*) in *O. sativa* was conducted by AlphaFold (Figure 6B). The results showed that *Os11g33394.1* is a transmembrane protein composed of two subunits consisting of twenty α-helices, five β-sheets, and several random coils structures. According to the prediction results of the tertiary structure, the *Os11G33394.1* gene has a α-helix containing 28 amino acid residues at the C-terminal, which constitutes the transmembrane domain of the protein. Motif 6 in the *SOFF* protein family has a total of 21 amino acid residues, which contains 5 non-polar and hydrophobic Ala and Leu, corresponding to the main body of the transmembrane domain (Figure 6A). In addition, according to P2RANK prediction results, the *Os11G33394.1* gene transmembrane structure predicted a ligand (base) binding pocket consisting of four residues at the C-terminal of α-helices. A short sequence containing only six amino acid residues downstream of the transmembrane structure of *Os11G33394.1* constitutes the intracellular part of the *SOFF* protein. Therefore, the main structure of *SOFF* protein consists of a non-cytoplasmic part, which is predicted to contain six ligand binding pockets (Figure 6B).

Based on the prediction results of the TMHMM transmembrane domain, most *SOFF* genes, including *Os11G33394.1*, had a transmembrane domain of about 23 amino acid residues at the C-terminal region. In contrast, there were still 39 genes in the SOFF family whose transmembrane domain had not been detected (Figure 5). Therefore, *SOFF* genes could be divided into two types: those with transmembrane domains at the C-terminal and those without or with partial transmembrane domains at the C-terminal, including the genes encoded by *Aspofficinalis01.231* in asparagus. Due to the existence of the transmembrane domain, *SOFF* protein structure was correspondingly divided into three parts: the transmembrane domain imbedded in the cell membrane, the main part of the protein located on the outer side of the membrane with the inner side having only a short peptide segment. According to the prediction of the tertiary structure and transmembrane domain of the asparagus *SOFF* gene sequence (*Aspofficinalis01.231*), the C-terminal of the *SOFF* protein lost the transmembrane domain and its downstream intracellular peptide, which may alter the protein localization at the membrane, and thus damage its function (Figure 6B).

Furthermore, the *SyGI* orthologous genes in *A. thaliana* (*At3g04280.1*) contain five hydrophilic α-helices located on the outside of the barrel structure, and five hydrophobic β-sheets located on the inside of the barrel (Figure 5D). A total of 23 amino acid residues of Phe^68^-Arg^90^ form a β-sheet and an α-helix, which also corresponds to the main body of the conserved Motif 1 of *SyGI* homologous proteins. This sequence fragment includes the core amino acid residue Asp^74^, which is related to the phosphate group transduction system.

The tertiary structure of the kiwifruit *SyGI* gene (*Actinidia33667.1*) was modeled using *At3G04280.1* protein as the template (Figure 5E). The modeling results showed that the kiwifruit *SyGI* gene has a tertiary structure highly identical to that of *Arabidopsis At3G04280.1* protein, which has a folded “barrel structure” composed of five α-helices and five β-sheets. The “barrel mouth” contains an Asp^57^ residue that accepts phosphoryl groups, corresponding to Asp^74^ of *At3G04280.1* in *A. thaliana*, but one notable difference is that the kiwifruit *SyGI* sequence lacks the long tail at the N-terminal.

### 3.7. Cis-Regulatory Element Analysis of SOFFs and SyGIs in A. thaliana

**Figure 7:**
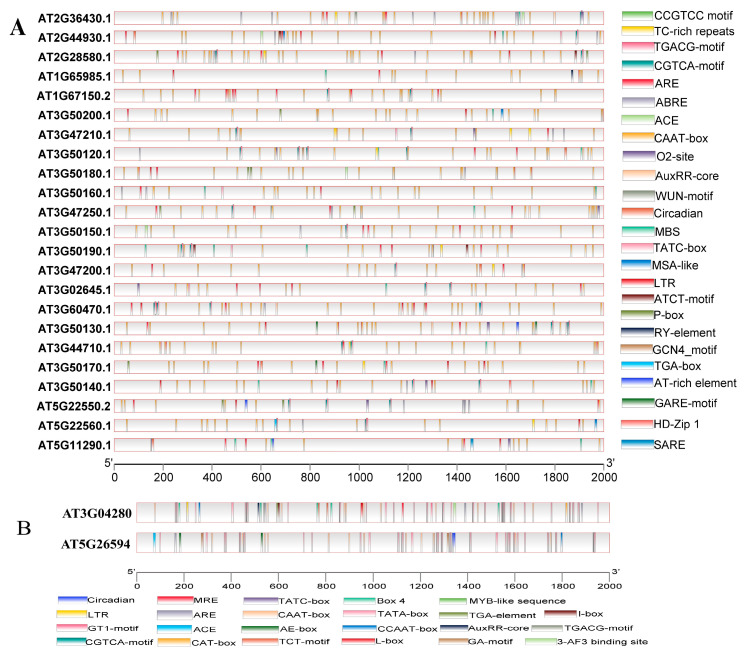
Promoter region analysis in *A. thaliana.* (**A**) *Cis*-regulatory elements in promoter region of *SOFF* genes. (**B**) *Cis*-regulatory elements in the promoter regions of *SyGI* genes.

Gene expression is usually regulated by *cis* elements in its upstream promoter sequence and its presence in the non-coding DNA region just before the transcription start site regulates various receptors or expression behaviors of genes in various environmental conditions [11]. Therefore, analyzing and exploring the regulatory elements of *Arabidopsis SOFF* and *SyGI* genes will improve our understanding of the regulatory mechanisms and their probable functions. To conduct predictive analysis of cis elements, a 2000 bp 5-flanking region upstream of the start codon of *SOFF* and *SyGI* genes in *Arabidopsis* was selected and analyzed using the PLANTCARE database. According to the results, both gene families had almost similar *cis*-regulatory elements in their respective promoter regions and were divided into several groups like growth and development, phytohormone responsive elements, abiotic stress responsive elements and other regulatory elements (Figure 7).

Promoter region analysis for *SOFF* and *SyGI* genes revealed that light-responsive elements accounted for 60% of the whole *cis*-regulatory elements involved in growth and development of *Arabidopsis*. Further regulatory elements related to the growth and development of plants were also identified including ACE, Box-II, G-box, Box-IV, LS7, Sp1, L-box, GTI-motif, GA-motif, I-box ATC-motif and AE-box, which are present in the promoter regions of *AT2G36430.1*, *AT2G36430.1*, *AT2G44930.1*, *AT1G67150.2*, *AT3G50180.1*, *AT3G50160.1*, *AT3G47250.1*, *AT3G04280.1* and *AT5G26594.1* (Table S3). Cis-regulatory elements related to metabolism regulation and plant development such as circadian rhythm control, cell cycle regulatory elements and O_2_ sites were identified in promoter regions of *AT3G47250.1*, *AT3G04280.1*, *AT3G02645.1*, *AT5G26594* and *AT3G50130.1. Cis* elements responsible for meristem expressions like CAT box are located in the promoters of *AT3G50160.1*, *AT3G04280.1* and *AT3G60470.1* and endospermic expression-related GCN4 is present in the promoter regions of *AT5G26594.1*, *AT3G60470.1* and *AT3G50120.1* (Figure 7B). However, some of the genes from *SOFF* and *SyGI* families have *cis* elements for seed specific regulation related to the RY-element in the promoter regions of *AT5G11290.1*. Therefore, we assumed that the GCN4 motif and RY-element may be involved in the regulation of plant endosperm and seed development and speculation that *SOFF* and *SyGI* genes may have a vital role in reproductive development of plants.

Plant growth hormones related to regulatory elements, including TGA-element, TGA-box and AuxRR-core (present in the promoter regions of *AT3G60470.1*, *AT3G50150.1*) involved in auxin responsiveness and salicylic acid responsive TCA elements, were identified in the promoter regions of several genes in both the families (Figure 7A and Tables S2 and S3). Similarly, methyl jasmonic acid-responsive elements (TGACG and CGTCA) and gibberellin-responsive elements such as TATC box and GARE-motif were identified in the promoter regions of AT*3G50150.1*, *AT3G50190.1*, *AT3G47200.1*, *AT5G26594.1*, *AT3G02645.1*, *AT3G44710.1*, *AT3G50170.1* and *AT3G04280.1*, respectively. Furthermore, abscisic acid-responsive *cis*-regulatory elements such as AAGAA and ABRE, and cytokinin-related cis elements such as G-box and B-box were identified in the promoter regions of *SOFF* and *SyGI* genes (*AT3G50140.1*, *AT3G04280.1*, *AT5G22550.2*, *AT5G22560.1*, *AT3G47210.1*).

Among the *cis*-regulatory elements for abiotic stresses, drought-responsive *cis* elements were in the majority and widely distributed in the promoter regions of *SOFF* and *SyGI* genes (Figure 7A,B). These cis elements include MBS, Myc, MYC, Myb and Myb binding sites responsible for drought and dehydration present in the promoter regions of *AT3G50200.1*, *AT2G44930.1*, *AT3G50140.1*, *AT3G50140.1*, *AT5G26594.1* and *AT3G04280.1*. Furthermore, some of these elements regarding defense and stress responses and low temperature stress such as TC-rich repeats, LTR and STRE were identified in the promoters of AT*3G50170.1*, *AT3G50130.1*, *AT3G44710.1*, *AT3G04280.1* and AT3G47200.1. Essential elements for anaerobic induction and hypoxia induction-responsive elements such as ARE (in the promoter region of *AT3G47200.1*, *AT3G50150.1* and *AT3G04280.1*) were observed, while some of the promoters (*AT3G47250.1*, *AT2G36430.1* and *AT5G26594.1*) had *cis*-regulatory elements (WUN-motif) for wound responsiveness and healing. Moreover, analysis revealed some other important *cis* elements including protein binding sites (ATBP-1), zein metabolism (O_2_ site) and common *cis* elements in promoter and enhancer regions (CAAT box, Tata box) that are widely present in both gene family’s promoter regions (*AT5G11290.1*, *AT5G22550.2*, *AT3G50170.1*, *AT2G36430.1*, *AT2G36430.1*, *AT2G44930.1*, *AT5G26594.1* and *AT3G04280.1*) (Tables S2 and S3).

### 3.8. STRING Protein Interaction and Functional Enrichment Analysis of SOFF and SyGI Genes in A. thaliana

**Figure 8:**
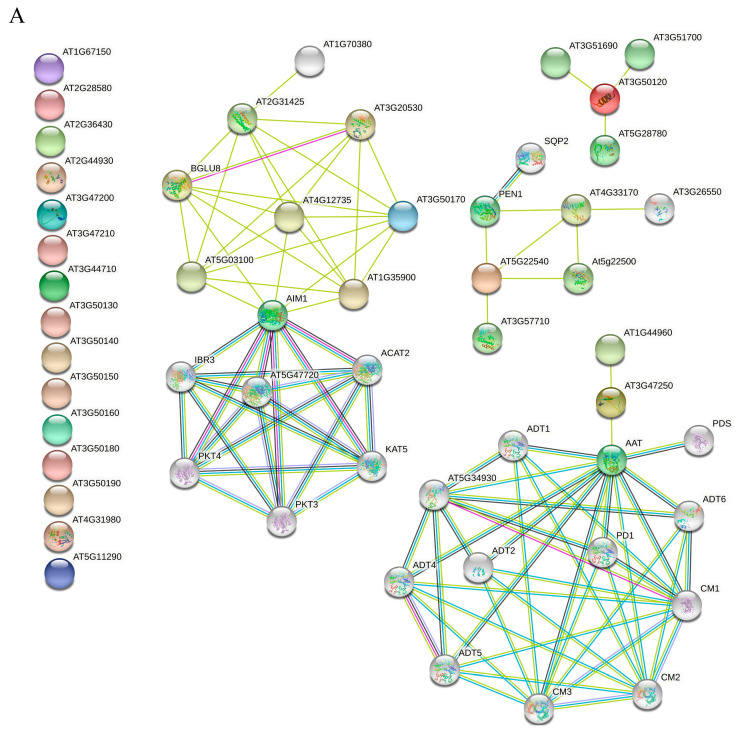

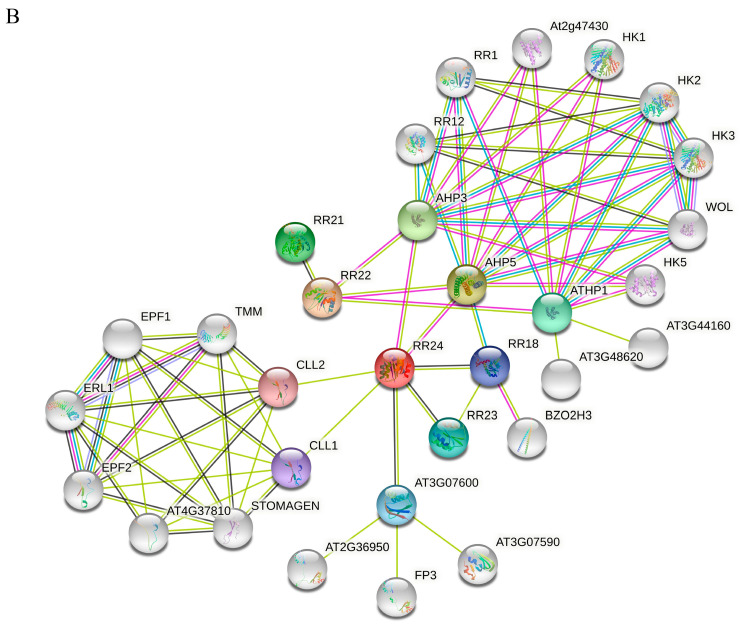
STRING regulatory networks analysis in *A. thaliana*. (**A**) Protein interaction of *SyGI* gene. (**B**) Protein interactions of *SOFF* gene in *A. thaliana*.

STRING protein interaction and functional enrichment analysis were performed on *SyGI* homologous genes *ARR22* and *ARR24* in *A. thaliana* to identify potential protein interactions or regulatory networks (Figure 8B). Phosphoric acid transfer response regulators, transcription factors, and cytokinin receptors were found to be linked to *ARR22* and *ARR24* genes. Biological processes such as phosphoric group transfer signal transduction, reproductive development, acid chemical response, secondary growth, antipodal cell differentiation, embryo sac differentiation, and so on were identified. In addition, RR-like proteins (*RR1*, *RR12*, *RR21*, *RR18*, *RR23*) and Histidine Phosphotransferase Proteins 1 *HP1*, *HP3*, *HP5* and Epidermal Patterning Factor (*EPF*) proteins (*EPF1*, *EPF2*, *CLL1*, *CLL2*, *AT4G37810.1*) were also detected in the regulatory network of ARR22 and *ARR24*. Special attention should be paid to HK (Histidine Kinase) proteins such as HK1, HK2, HK3, HK4 (WOL), and HK5 involved in plant hormone signaling (CK signal and ABA signal), displaying an extensive and close interaction with *RR22* and *ARR24* regulatory networks, which is consistent with the results of CLIME analysis, indicating that these proteins or genes not only have similar co-evolution characteristics but also have interactions or regulatory relationships in *A. thaliana*.

Similarly, 19 *SOFF* genes in *A. thaliana* were submitted to the STRING website for protein interaction and functional analysis (Figure 8A). Unfortunately, the results revealed that the 19 *SOFFs* did not have any direct protein interactions or regulation relationships, and only 4 genes were detected in the weak enrichment of regulation network relating to primary and secondary metabolites, biosynthesis of aromatic amino acids, and fatty acid degradation and other relevant functions.

## 4. Discussion

Kiwifruit *SyGI* genes belong to the cytokinin response regulator (RR) family in the cytokinin signaling pathway. As key transcription factors in cytokinin signal transduction, cytokinin response regulatory factors are usually divided into three categories according to their structure and function: Type-A RRs are induced by cytokinin and are usually used as marker genes in the cytokinin signal transduction pathway. Type-B RRs mainly act as a transcription factor to regulate type-A RRs, or interactors of other proteins to regulate growth and development and stress response. The structure of type-C RRs is like type-A, and their expressions are not affected by cytokinin [40]. In A. thaliana, *ARR22* acts as a phosphatase on specific AHPs to disrupt the phosphoryl group transfer in the two-component system (TCS) and prevent the phosphorylation of Type-B ARRs, thus blocking the activation of Type-B RR genes [41].

The Type-C RR *ARR22/24* in the *A. thaliana* RR family is an orthologous protein of kiwifruit *SyGI* protein. We performed the evolutionary analysis of these proteins, which showed that *SyGI* genes were formed from the gene replication before 20MYRs. During the evolutionary process, one copy gradually gained a new function [42] and was expressed specifically on the epidermis of the immature carpel of male flowers to inhibit the female functions, which resulted in the sex control of kiwifruit [5,11]. The *PopARR17* gene in poplar of perennial dioecious species functions as a sex switch, triggering female development when turned on, and stimulating male development when it is turned off [43]. These results are consistent with our phylogenetic analysis that shows that the *SOFF* gene family exists in major biological species such as prokaryotes, protists, plants, fungi, and animals, proving the importance and universality of their functions in organisms. However, *SyGI* orthologous genes were lost in some lineages or species of protists and animals, and their important functions were replaced by other homologous genes in the RR family. Interestingly, the current phylogenetic analysis displayed that the *SOFF* gene family evolved mainly with WGD/segmental duplication and was divided into two subfamilies and seven groups (Figure 1). On the other hand, the *SyGI* protein family is smaller with compact molecules and was divided into three groups (Figure 2). These results suggest that both the gene families showed similar evolutionary patterns with a minor exception. The functional divergence may be more significant in the *SOFF* gene family as compared to the *SyGI* gene family due to their large size and the duplicated gene pair involvement in diversifying selection. Furthermore, the Evolutionarily Conserved Module (ECM) analysis detected 19 *SOFF* genes in *Arabidopsis*, and these were divided into three ECMs, where ECM1 and ECM2 homologous genes exist only in plants. ECM1 orthologs originally existed in the plant ancestor moss, while ECM2 orthologous genes were found in monocots and dicots. Orthologs in ECM3 are ubiquitous in protists, plants, fungi, and metazoans (Figure 3). In addition, 2 *SyGI* genes in *A. thaliana* were identified as the same ECM as the other 42 genes, most of which are involved in cytokinin signal transduction and widely exist in plants and animals. The fact that ECM1 and ECM2 homologous genes are only found in plants emphasizes their unique functions in processes unique to plants. These genes may have arisen early in plant evolution and are linked to basic characteristics needed for terrestrial adaption, such as desiccation resistance or basic tissue organization, as shown by the discovery of ECM1 orthologs in the ancient moss lineage [44]. Following the split of angiosperms, ECM2 genes, which are present in both monocots and dicots, suggest additional functional diversity that may be connected to the development of intricate reproductive structures or vascular systems [45]. The biochemical pathways represented by the ECM3 genes most likely existed before the main eukaryotic lineages diverged. Their persistence throughout kingdoms suggests that they play a crucial role in basic biological processes, which may be connected to signaling networks, cell division, or stress reactions [46].

Furthermore, synteny analysis revealed that 125 duplicated gene pairs were identified in Kiwifruit where WGD/segmental duplication played a major role in duplicating these events (Figure 4A). However, no syntenic relations were observed in the asparagus genome due to its small genome size and un-sequenced member of its family. Scientists believe that several whole-genome duplications are the primary cause of the high degree of expansion of the gene family [44]. In many other angiosperms, WGD events have fueled genome growth and functional diversity, which is in line with the kiwifruit genome’s preponderance of WGD and segmental duplications. Synteny signals may be obscured by significant genomic rearrangements or deletion of duplicate genes that frequently occur in small genomes [47]. They may also result in gene functional redundancy while encouraging the expansion of eukaryotic gene families [48].

Up to now, the research and understanding of the function of genes containing the DUF247 domain are very limited. Our results confirmed that the *SOFF* gene containing the DUF247 domain located in the non-recombination region of Y chromosome in asparagus had a female inhibitory function [16]. The DUF247-containing gene *LpSDUF247* in ryegrass is the male determinant of self-incompatibility of multiple S alleles [49], while the *LpSDUF247* homolog *OlSS1* in *Oryza longistaminata* is expressed specifically in stamens. *OlSS2* is expressed in both stamens and pistils and may also be involved in the control of self-incompatibility of *O. longistaminata* [50]. According to the findings of the conserved motif analysis, three motifs are present in every member of *SOFF* and *SyGI* (Figure S1 and Figure 6), whilst some other motifs are unique to specific genes in both families. The functional differentiation of members of the *SyGI* and *SOFF* gene families are largely due to these distinctive motif patterns [51]. Furthermore, the secondary structure of the proteins from the *SOFF* and *SyGI* gene families differed in the proportions of α-helices, extended chains, irregular coils, and β-turns (Figure 5 and Figure 6). This leads to variations in the spatial folding of the proteins, supporting the functional differences and evolutionary expansion of the two gene family members. Previous studies have suggested that the secondary structure of a gene influences how a protein interacts with other molecules, such as in the case of enzymes binding to substrates or inhibitors [52]. Experiments on promoter regions speculated that *cis* elements in the upstream promoter region of a gene often control its expression [53]. These *cis* elements, which are found in non-coding DNA upstream of the gene transcription start site, control how genes express themselves under various conditions, including stress or tissue-specific expressions [54]. Consequently, it is possible to comprehend the regulatory processes of the *SOFF* and *SyGI* gene families and forecast their possible functions by analyzing the *cis* elements implicated in their regulation. At present, the promoter region of both *A. thaliana* gene families contains many *cis* elements linked to growth, development, stress, and phytohormone responses, the majority of which are engaged in plant growth and reproduction (Figure 7), thus providing clear evidence that these genes may have roles in sex determination and regulating the reproduction mechanisms [55].

In addition, protein interaction analysis of the *SyGI* genes showed that most of the 42 genes with similar evolutionary characteristics to the *ARR22* and *ARR24* gene in *A. thaliana* were related to cytokinin signal transduction, such as the *ARR* gene family, *APRR* (two-component response regulator, pseudo-response regulator) gene, *HK* (*Histidine kinase*) gene family, and some other genes related to ethylene signal transduction, indicating these findings are consistent with those of CLIME analysis (Figure 8). As essential plant functions like cell division, differentiation, and stress responses are regulated by cytokinin [56]. Therefore, the conserved connections suggest that the *SyGI* gene family has evolved to play a role in regulating signal transduction, which may have an impact on plant development and stress tolerance [57]. Furthermore, the *SOFF* gene interaction network in *A. thaliana* is surprising because many of the *SOFF* genes failed to identify any interactions or regulatory networks. This absence of interactions may indicate their specialized functions, redundancy, or reliance on specific environmental or developmental conditions for activation. However, only four *SOFF* gene family members were associated with secondary metabolites, biosynthesis of aromatic amino acids, and fatty acid degradation, and other functions. These pathways are essential for plant growth, stress responses, and metabolic regulation [58]. The relevance of *SOFF* genes in fatty acid breakdown and secondary metabolites suggests that they may play a part in energy metabolism and plant defense mechanisms. The absence of more extensive networks of interactions may be a result of their roles in *A. thaliana* not being fully described [59]. Future studies focusing on transcriptomics, proteomics, and metabolomics could elucidate their specific regulatory mechanisms.

Both *SOFF* and *SyGI* gene families are equally important in monoecious and diecious species for their roles in sex determination. *SyGI* is an essential factor in kiwifruit (*A. chinensis*) and persimmon (*Diospyros kaki*) sex determination by working as a female gene and suppressing male development through epigenetic regulation [60]. However, the *SOFF* gene family in asparagus (*A. officinalis*) plays a crucial role in sex differentiation by countering feminizing pathways and is involved in male-specific expression pathways [15]. The results of these discoveries show a simultaneous evolution of sex determination systems and point to a wider conservation of these genetic pathways throughout plant lineages. Additionally, both gene families may have role in other developmental and physiological processes in plants because evidence suggests that sex determinant genes often display pleiotropic effects like reproductive development, regulating hormonal pathways and stress responses [61]. Specifically, gibberellin signaling, a vital phytohormone pathway involved in both general growth and reproductive organ development regulation, may be influenced by *SOFF* and *SyGI* genes [62], which further demonstrate that they might be involved in broader plant developmental processes. Furthermore, utilizing SOFF and SyGI genes offers sex expression regulation and novel hybrid breeding strategies for improved yields. Manipulation of these genes will lead us to selective propagation of desirable sex phenotypes with increased quality and yield in dioecious crops like asparagus, kiwifruit and persimmon [63]. Also, the way they may interact with epigenetic regulatory networks provides opportunities for precision breeding using RNA interference and CRISPR techniques, which enable targeted alterations to improve crop yield and adaptability. These scenarios highlight the value of bioinformatics-based research in understanding the genetic basis of sex determination and its wider plant breeding implications.

## 5. Conclusions

This study provides a comprehensive bioinformatics analysis of the *SOFF* and *SyGI* gene families, revealing key insights into their evolutionary relationships, structural characteristics, and functional potential in plant sex determination. Our classification divided *SOFF* into two families with seven distinctive groups, while *SyGI* formed three groups, highlighting the diversity within these gene families. ECM analysis placed two *SyGI* genes in a group with forty-two others, suggesting potential functional associations. Synteny analysis identified 125 duplicate gene pairs in kiwifruit, indicating extensive gene duplication events that may have contributed to the diversification and retention of SyGI genes. Structurally, *SOFF* contains a DUF247 domain, while SyGI features a Rec-like barrel-shaped domain with five alpha and beta strands, suggesting distinct structural adaptations that might undermine their functional roles. The presence of diverse *cis*-regulatory elements suggests participation in complex transcriptional regulation. Protein interaction analysis revealed four close interactions for *SOFF* genes, whereas *SyGI* exhibited extensive interactions, indicating a broader regulatory role. These findings collectively highlight the evolutionary divergence and functional specialization of *SOFF* and *SyGI* in plant reproductive biology and provide valuable bioinformatics insights into the genetic architecture of both gene families, which lay a foundation for future functional studies and potential breeding applications in asparagus and kiwifruit, particularly for sex-specific selection, hybrid breeding, and improving reproductive traits. Moving forward, experimental validation through gene expression profiling and functional assays will be essential to further substantiate the bioinformatics predictions and unravel the precise regulatory roles of these genes in plant reproductive development.

## Data Availability

The original contributions presented in this study are included in the article/Supplementary Materials. Further inquiries can be directed at the corresponding authors.

## Supplementary Materials

The following supporting information can be downloaded at https://www.mdpi.com/article/10.3390/genes16030280/s1; File S1: Output results of CLIME (CLustering by Inferred Models of Evolution); File S2: Output results of CLIME (CLustering by Inferred Models of Evolution); Figure S1: Comparison of the conserved domain and motifs in SOFF genes. (A) The NJ phylogenetic tree was constructed based on the full-length sequences of SOFF genes using MEGA 10.2 software. (B) The motif composition of SOFF genes. (C) Schematic representation of the conserved domains in SOFF proteins. The SOFF TM (transmembrane) domains are highlighted by light brown boxes, and the other domains are shown in the legend, respectively (D). The length of protein can be estimated using the scale at the bottom; Table S1: (a) SOFF gene information; (b) SyGI gene information; Table S2: Promotor region of SOFF genes in Arabidopsis; Table S3: Promotor region of SyGI genes in Arabidopsis; Table S4: KA KS values SyGI gene pairs in Kiwi fruit.
