# Supplementary material for: Insight into the Characterization of Two Female Suppressor Gene Families: SOFF and SyGI in Plants
Source: Genes (Basel). 2025 Feb 26;16(3):280. doi: 10.3390/genes16030280 (PMC11941796; doi:10.3390/genes16030280)
Supplement: Supplementary file 1 [file genes-16-00280-s001.zip › File S1.pdf]

Output results of CLIME (CLustering by Inferred Models of Evolution)

Dataset:

Num of genes in input gene set: 19
Total number of genes: 27361
Prediction LLR threshold: 0

The CLIME PDF output two sections:

1) Overview of Evolutionarily Conserved Modules (ECMs)

- Top panel shows the predefined species tree.
- Bottom panel shows the partition of input genes into Evolutionary Conserved Modules (ECMs), ordered by ECM strength (shown at right), and separated by horizontal lines.
- Each row show one gene, where the phylogenetic profile indicates presence (blue) or absence (gray) of homologs in each species (column).
- Gene symbols are shown at left. Gray color indicates that the gene is a paralog to a higher scoring gene within the same ECM (based on BLASTP E < 1e-3).

2) Details of each ECM and its expansion ECM+

- Top panel shows the inferred evolutionary history on the predefined species tree. Branch color shows the gain event (blue) and loss events (red color, with brighter color indicating higher confidence in loss). Branches before the gain or after a loss are shown in gray.
- Bottom panel shows the input genes that are within the ECM (blue/white rows) as well as all genes in the expanded ECM+ (green/gray rows). The ECM+ includes genes likely to have arisen under the inferred model of evolution relative to a background model, and scored using a log likelihood ratio (LLR).
- PG indicates "paralog group" and are labeled alphabetically (i.e., A, B). The first gene within each paralog group is shown in black color. All other genes sharing sequence similarity (BLAST E < 1e-3) are assigned to the same PG label and displayed in gray.

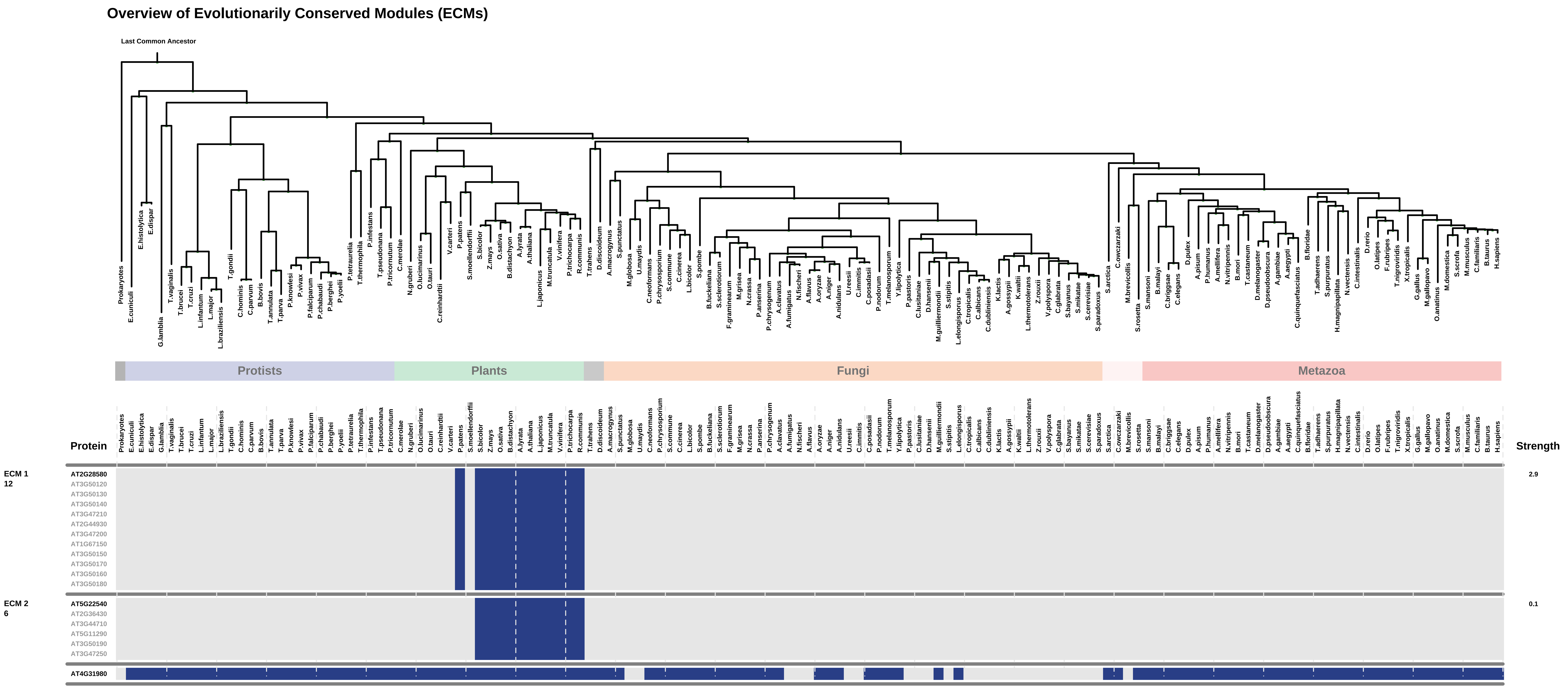
Overview of Evolutionarily Conserved Modules (ECMs)
Last Common Ancestor
Protists
Plants
Fungi
Metazoa
Protein
Strength
ECM 1
12
AT2G28580
AT3G50120
AT3G50130
AT3G50140
AT3G47210
AT2G44930
AT3G47200
AT1G67150
AT3G50150
AT3G50170
AT3G50160
AT3G50180
2.9
ECM 2
6
AT5G22540
AT2G36430
AT3G44710
AT5G11290
AT3G50190
AT3G47250
0.1
AT4G31980

ECM 1, Page 1

Num of ECM Genes: 12. Num of Predicted Genes: 200. ECM Strength: 2.9

PRESENCE — ABSENCE — GAIN — LOSS

Log-likelihood Ratio Scale: 0 10 20 30 40 50 60

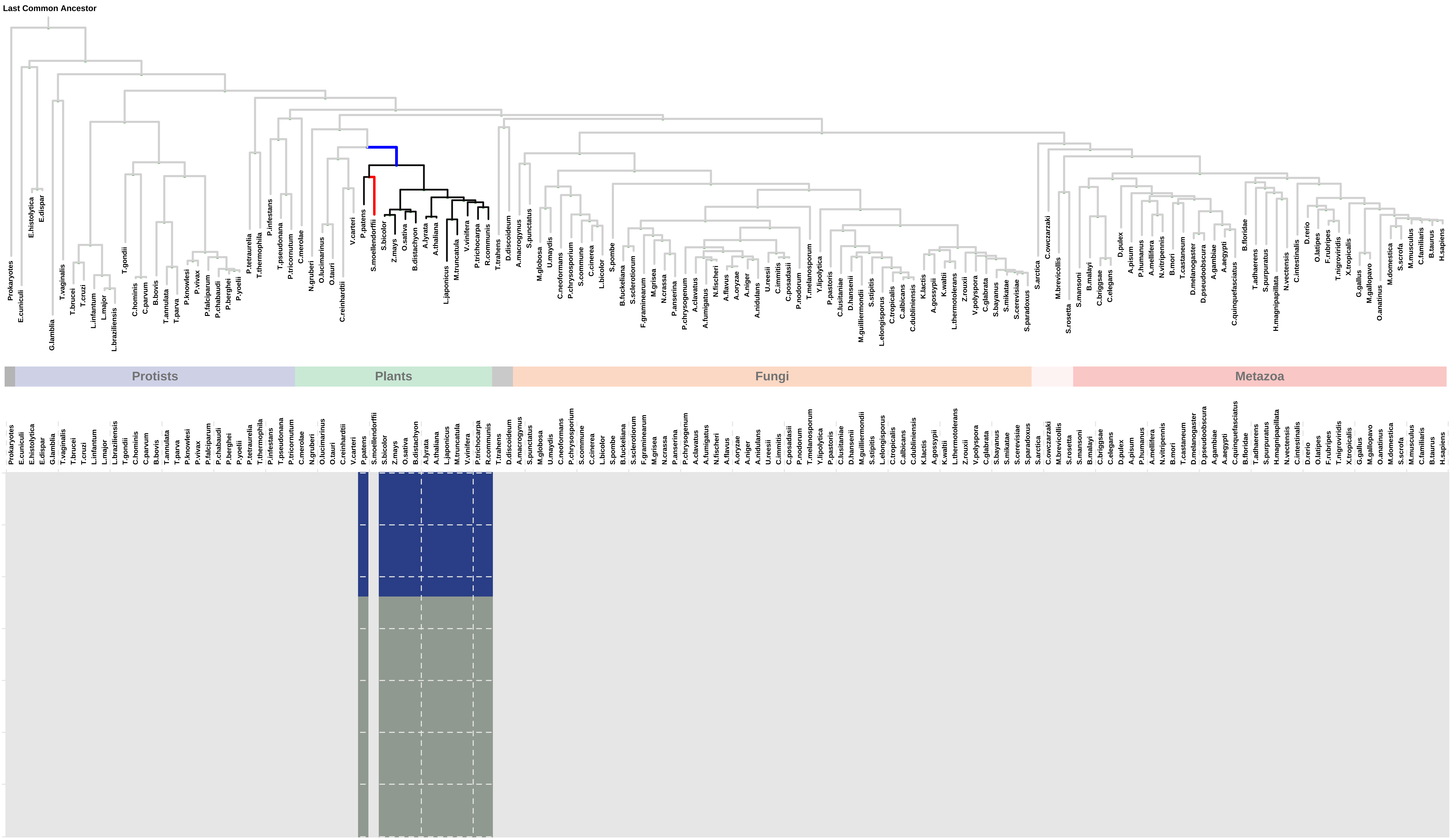

PG	Protein	LLR	Notes
A	AT2G28580		
A	AT3G50120		
A	AT3G50130		
A	AT3G50140		
A	AT3G47210		
A	AT2G44930		
A	AT3G47200		
A	AT1G67150		
A	AT3G50150		
A	AT3G50170		
A	AT3G50160		
A	AT3G50180		
B	GG1	4.0	
	AT4G08240	4.0	
C	AT4G15070	4.0	
	AT4G02090	4.0	
	AT4G01360	4.0	
C	AT4G01930	4.0	
D	AT1G43667	4.0	
E	AT1G56020	4.0	
	AT1G62790	4.0	
	AT1G65032	4.0	
F	AT4G11655	4.0	
G	AT5G57720	4.0	
	MPS1	4.0	
	AT5G51790	4.0	
H	AT5G49850	4.0	
	AT2G30505	4.0	
G	AT1G49475	4.0	
	AT1G15010	4.0	
	KDR	4.0	
H	AT5G49860	4.0	
I	AT1G31090	4.0	
J	AT3G54570	4.0	
I	AT3G21130	4.0	

ECM 1, Page 2

Num of ECM Genes: 12. Num of Predicted Genes: 200. ECM Strength: 2.9

PRESENCE — ABSENCE — GAIN — LOSS —

Log-likelihood Ratio Scale: 0 10 20 30 40 50 60

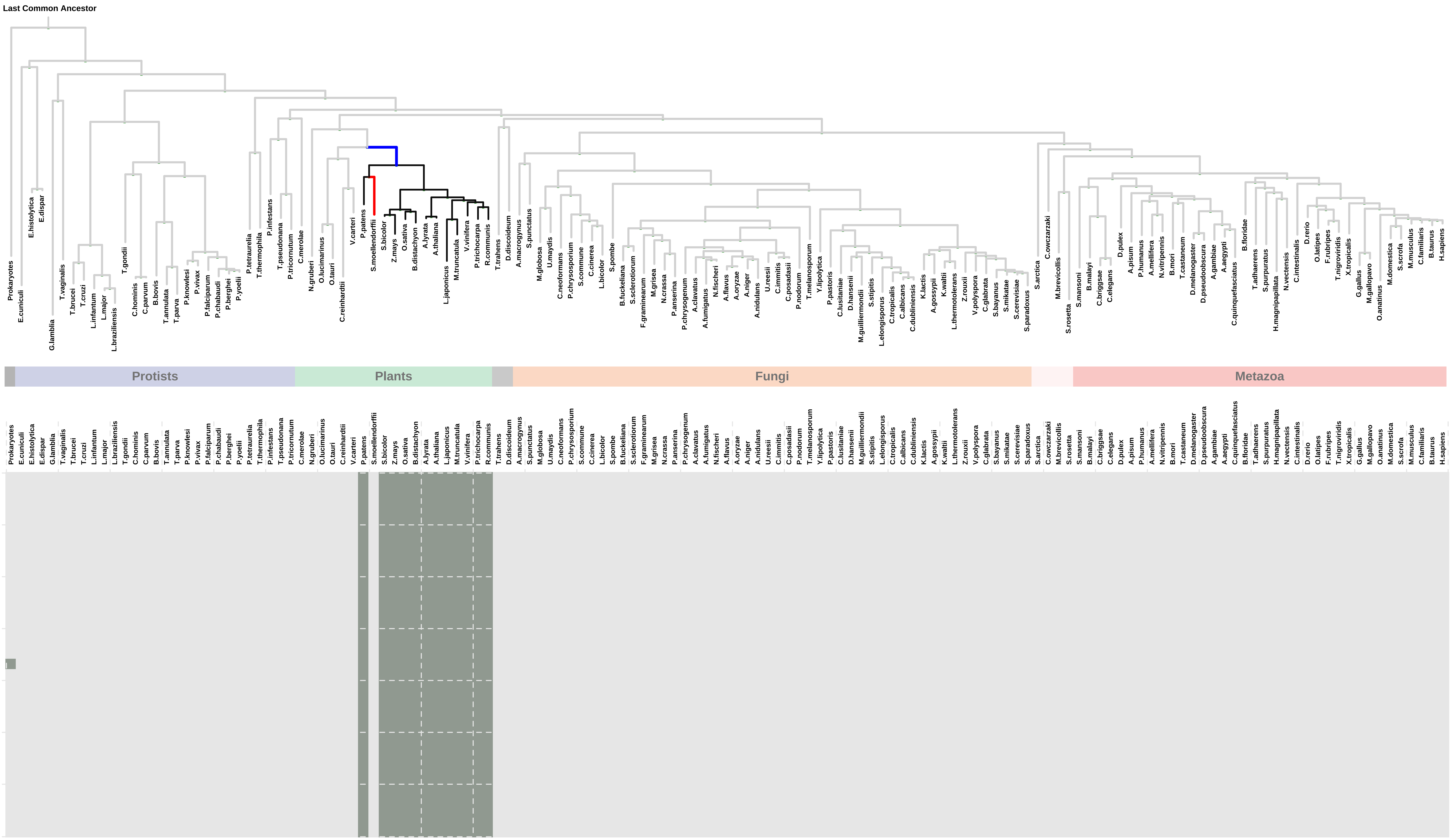

PG	Protein	LLR	Notes
I	AT3G21410	4.0	
	AT3G23740	4.0	
	AT3G23910	4.0	
I	AT3G25550	4.0	
C	AT3G26550	4.0	
K	AT3G52790	4.0	
L	ROH1	4.0	
M	COR47	4.0	
N	AT1G20520	4.0	
L	AT1G43630	4.0	
I	AT3G19470	4.0	
I	AT1G46840	4.0	
I	AT1G46984	4.0	
O	AT3G56410	4.0	
	AT3G49890	4.0	
M	ERD10	4.0	
L	AT1G18740	4.0	
I	AT1G67130	4.0	
P	AT1G29820	4.0	
Q	AT1G69325	4.0	
R	AT5G54300	4.0	
G	AT3G17010	4.0	
I	AT3G17560	4.0	
	AT5G54855	4.0	
C	AT2G28460	4.0	
S	AT3G50800	4.0	
C	AT4G01920	4.0	
C	AT4G01925	4.0	
	AT3G30200	4.0	
	AT4G20970	4.0	
I	CEG	4.0	
I	AT3G22730	4.0	
B	AGG2	4.0	
I	AT3G47150	4.0	
	AT2G39530	4.0	

ECM 1, Page 3

Num of ECM Genes: 12. Num of Predicted Genes: 200. ECM Strength: 2.9

PRESENCE — ABSENCE

GAIN — LOSS

Log-likelihood Ratio Scale

0 10 20 30 40 50 60

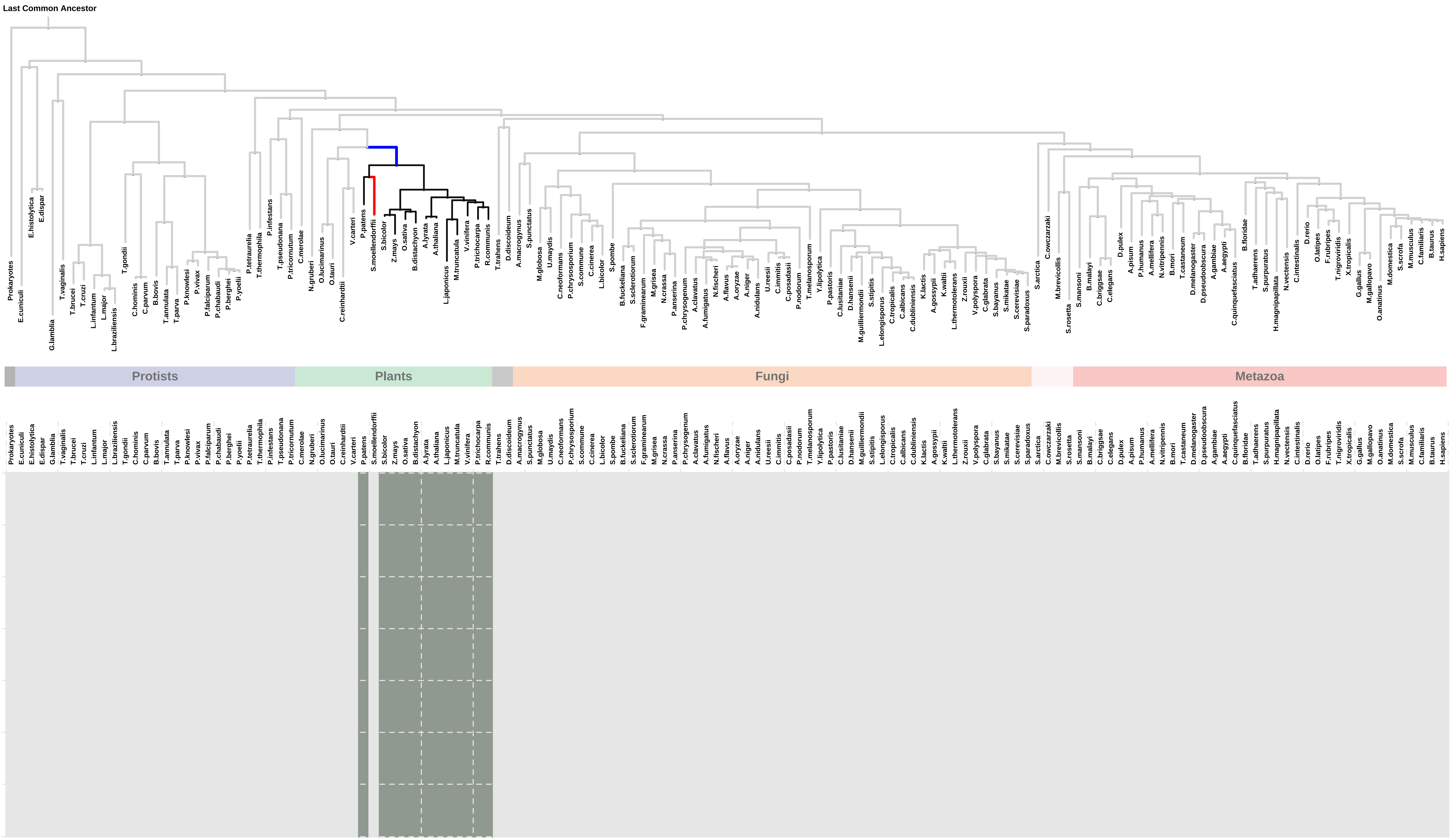

PG	Protein	LLR	Notes
I	AT2G34280	4.0	
T	LTP2	4.0	
T	LP1	4.0	
C	AT4G10370	4.0	
Q	AT1G13920	4.0	
	AT5G28040	4.0	
C	AT5G22355	4.0	
	AT4G17440	4.0	
I	AT1G53360	4.0	
I	AT1G53815	4.0	
M	ERD14	4.0	
	AT1G76705	4.0	
G	AT4G31640	4.0	
C	AT1G53340	4.0	
I	AT1G47800	4.0	
	AT1G06980	4.0	
R	AT1G61260	4.0	
C	AT1G55440	4.0	
	AT1G71780	4.0	
G	AT4G31680	4.0	
G	AT1G16640	4.0	
F	AT3G55390	4.0	
S	AT1G28190	4.0	
	AT2G40130	4.0	
I	AT4G17780	4.0	
K	AT4G25433	4.0	
G	AT4G31650	4.0	
	AT4G35980	4.0	
	AT4G39235	4.0	
	AT2G24692	4.0	
	AT1G55205	4.0	
C	AT1G55430	4.0	
	AT1G73940	4.0	
G	AT4G31615	4.0	
L	AT4G23530	4.0	

ECM 1, Page 4

Num of ECM Genes: 12. Num of Predicted Genes: 200. ECM Strength: 2.9

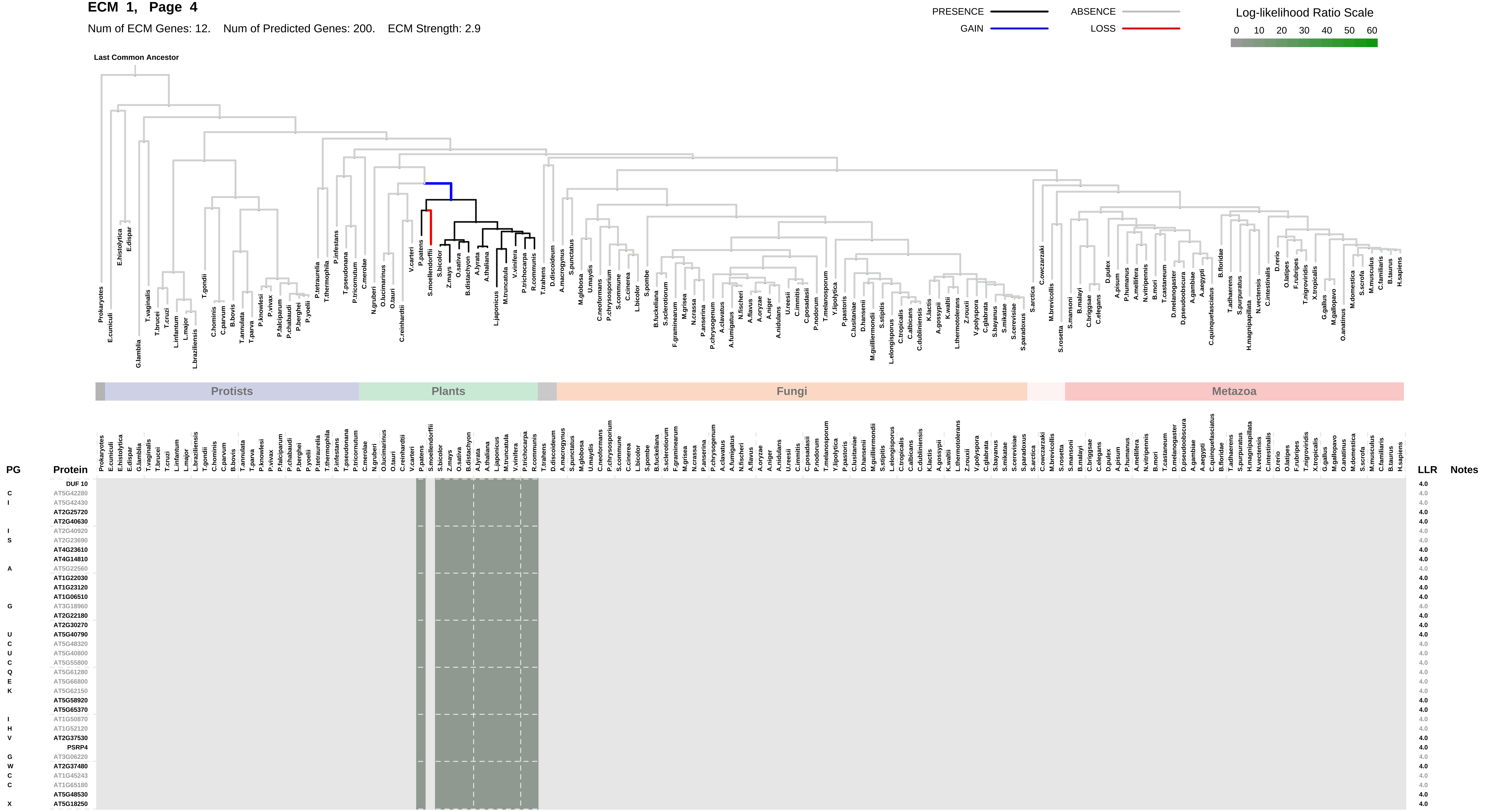

ECM 1, Page 5

Num of ECM Genes: 12. Num of Predicted Genes: 200. ECM Strength: 2.9

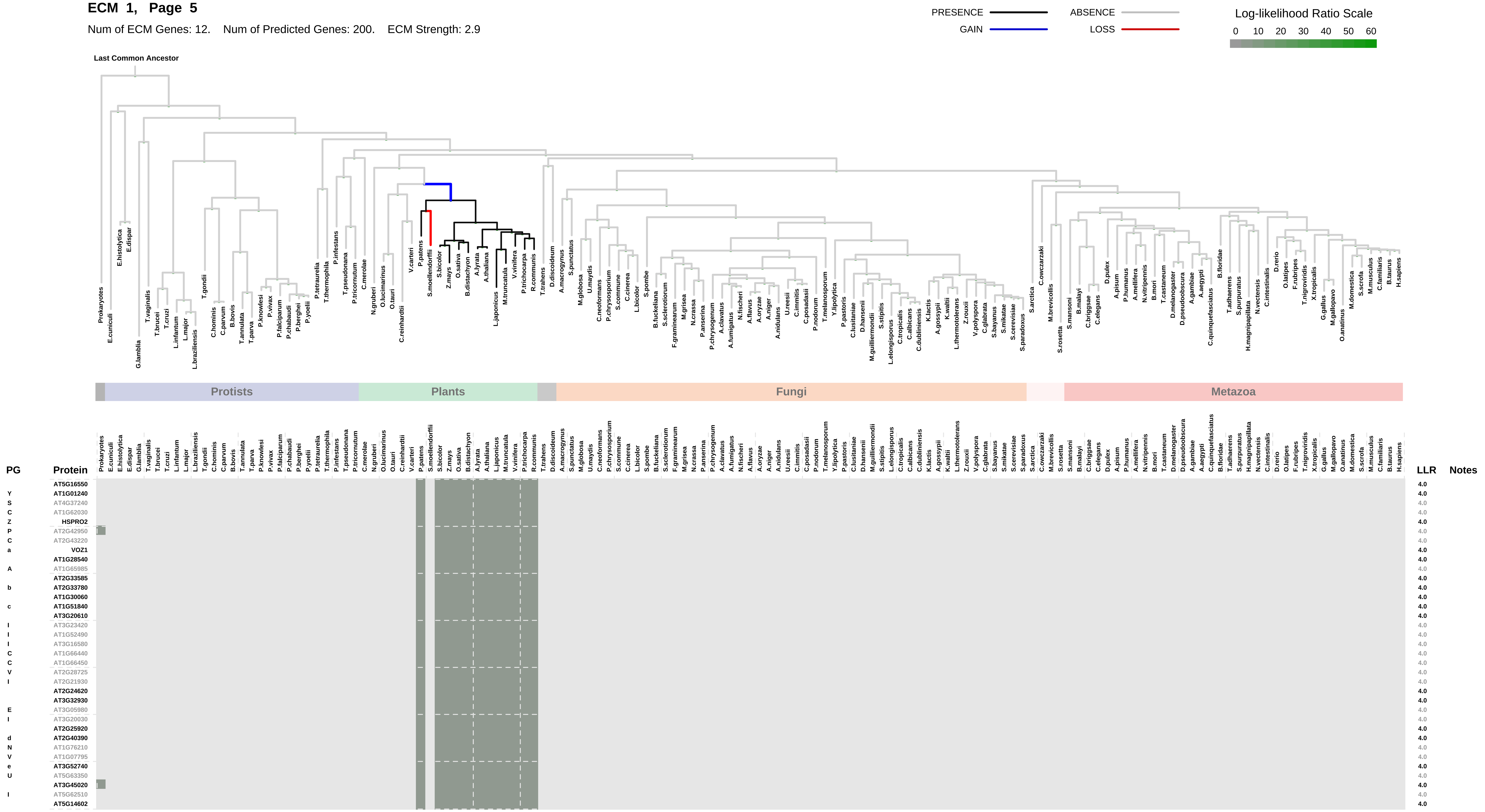

ECM 1, Page 6

Num of ECM Genes: 12. Num of Predicted Genes: 200. ECM Strength: 2.9

PRESENCE — ABSENCE — GAIN — LOSS

Log-likelihood Ratio Scale: 0 10 20 30 40 50 60

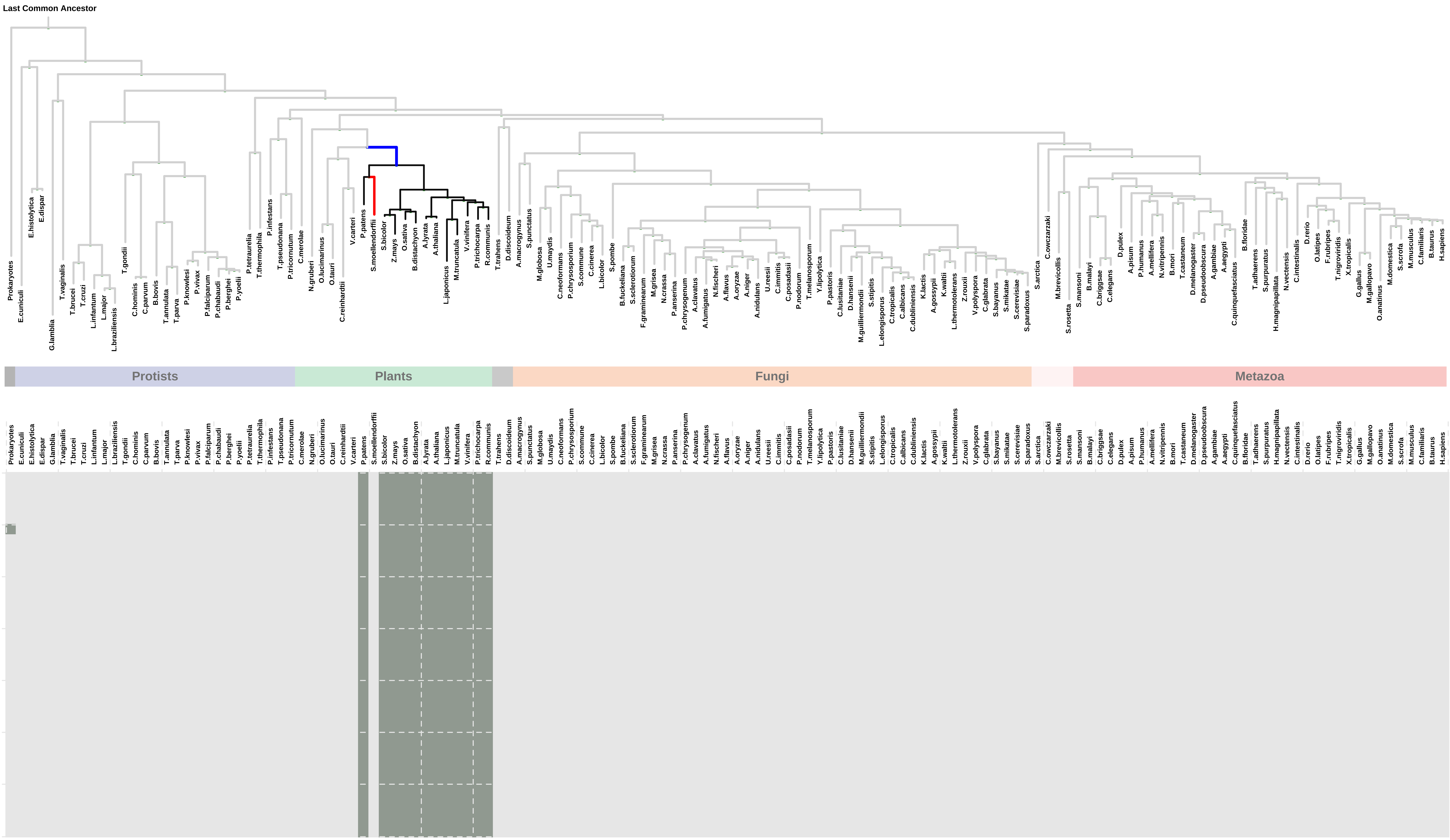

PG	Protein	LLR	Notes
I	AT5G62660	4.0	
C	AT3G45530	4.0	
c	AT3G46240	4.0	
d	AT5G64190	4.0	
T	LTP4	4.0	
	AT3G52905	4.0	
W	AT3G53670	4.0	
D	AT3G18280	4.0	
	AT3G13010	4.0	
	AT5G15480	4.0	
	AT2G41950	4.0	
I	AT4G19940	4.0	
O	AT5G05190	4.0	
Z	AT3G55840	4.0	
Y	AT2G46550	4.0	
L	AT4G11300	4.0	
	AT4G17960	4.0	
J	AT5G04020	4.0	
b	AT5G53830	4.0	
f	AT2G45900	4.0	
b	AT3G15300	4.0	
	SDE5	4.0	
e	AT3G44450	4.0	
	AT3G51010	4.0	
T	LTP5	4.0	
f	AT3G61380	4.0	
	AT5G55210	4.0	
	AT3G11810	4.0	
	AT2G24340	4.0	
	RALFL32	4.0	
X	AT2G41945	4.0	
a	VOZ2	4.0	
I	AT4G10740	4.0	
G	AT4G01580	4.0	
X	AT3G04040	4.0	

ECM 1, Page 7

Num of ECM Genes: 12. Num of Predicted Genes: 200. ECM Strength: 2.9

PRESENCE — ABSENCE — GAIN — LOSS

Log-likelihood Ratio Scale: 0 10 20 30 40 50 60

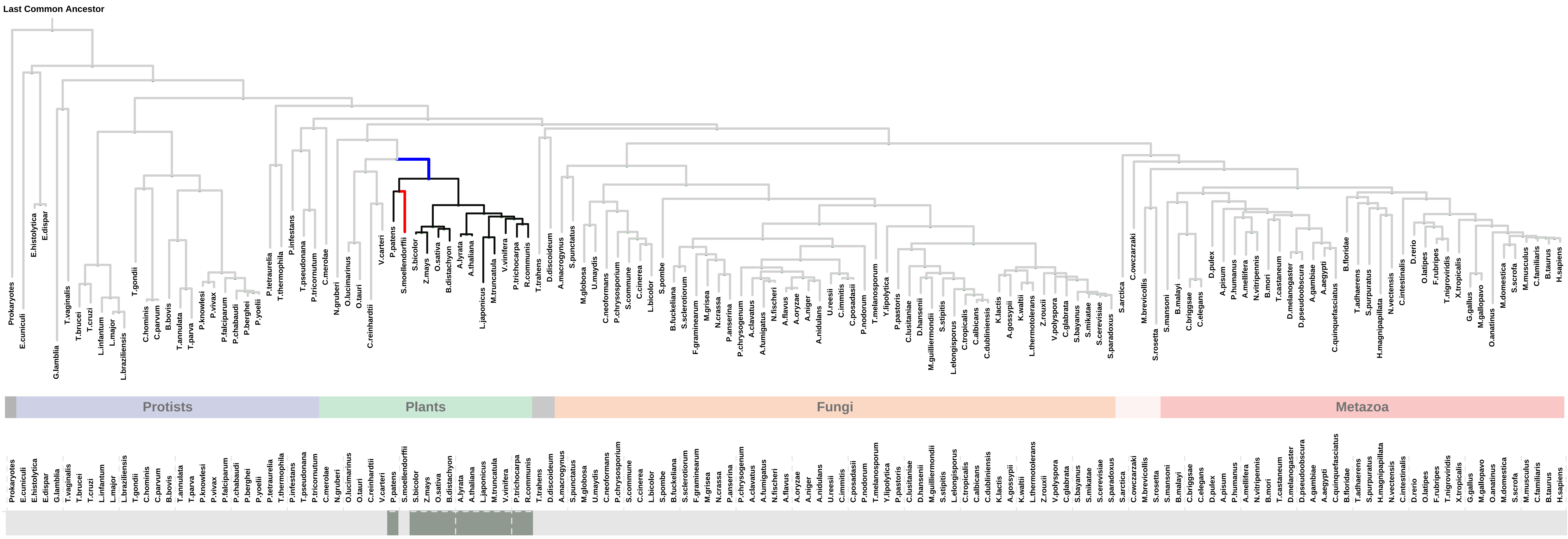

PG	Protein	LLR	Notes
S	CIVIF2	4.0	
	AT5G66580	4.0	

ECM 2, Page 1

Num of ECM Genes: 6. Num of Predicted Genes: 0. ECM Strength: 0.1

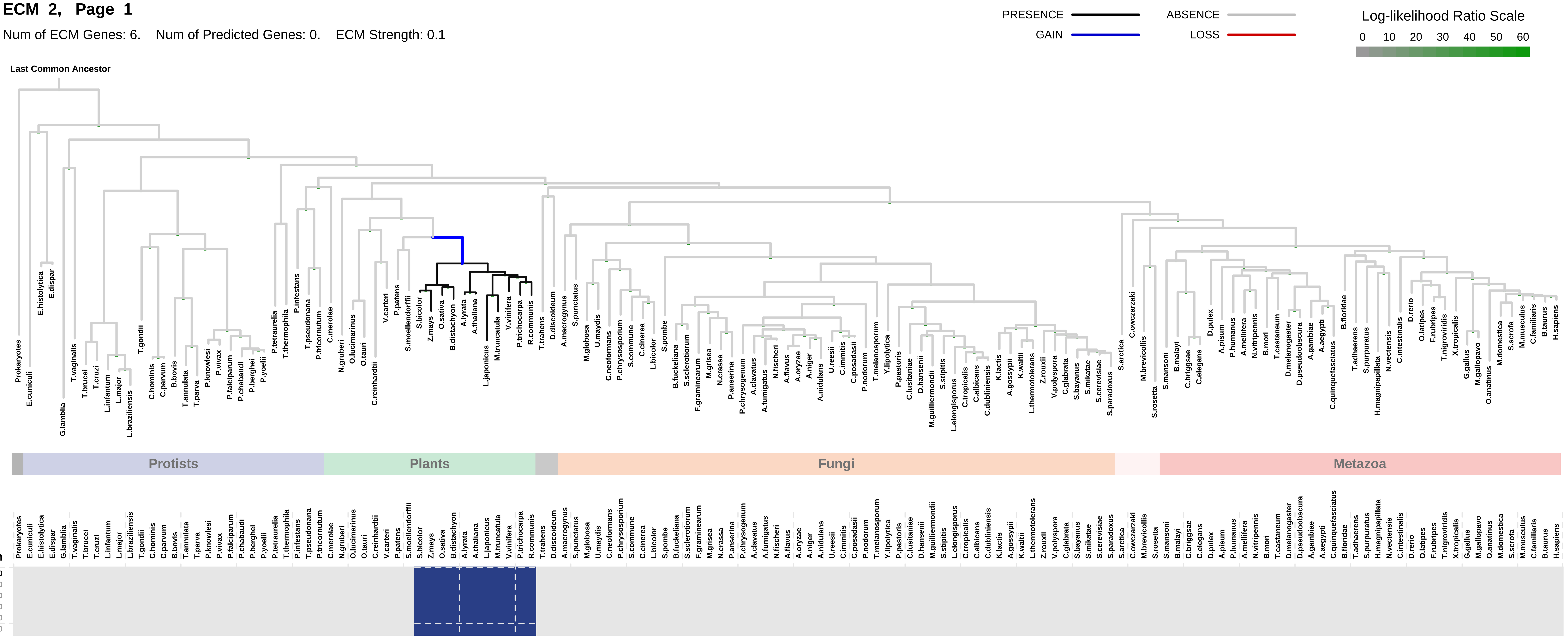

PG	Protein	LLR	Notes
A	AT5G22540		
A	AT2G36430		
A	AT3G44710		
A	AT5G11290		
A	AT3G50190		
A	AT3G47250		

ECM 3, Page 1

Num of ECM Genes: 1. Num of Predicted Genes: 21

PRESENCE — ABSENCE — GAIN — LOSS

Log-likelihood Ratio Scale: 0 10 20 30 40 50 60

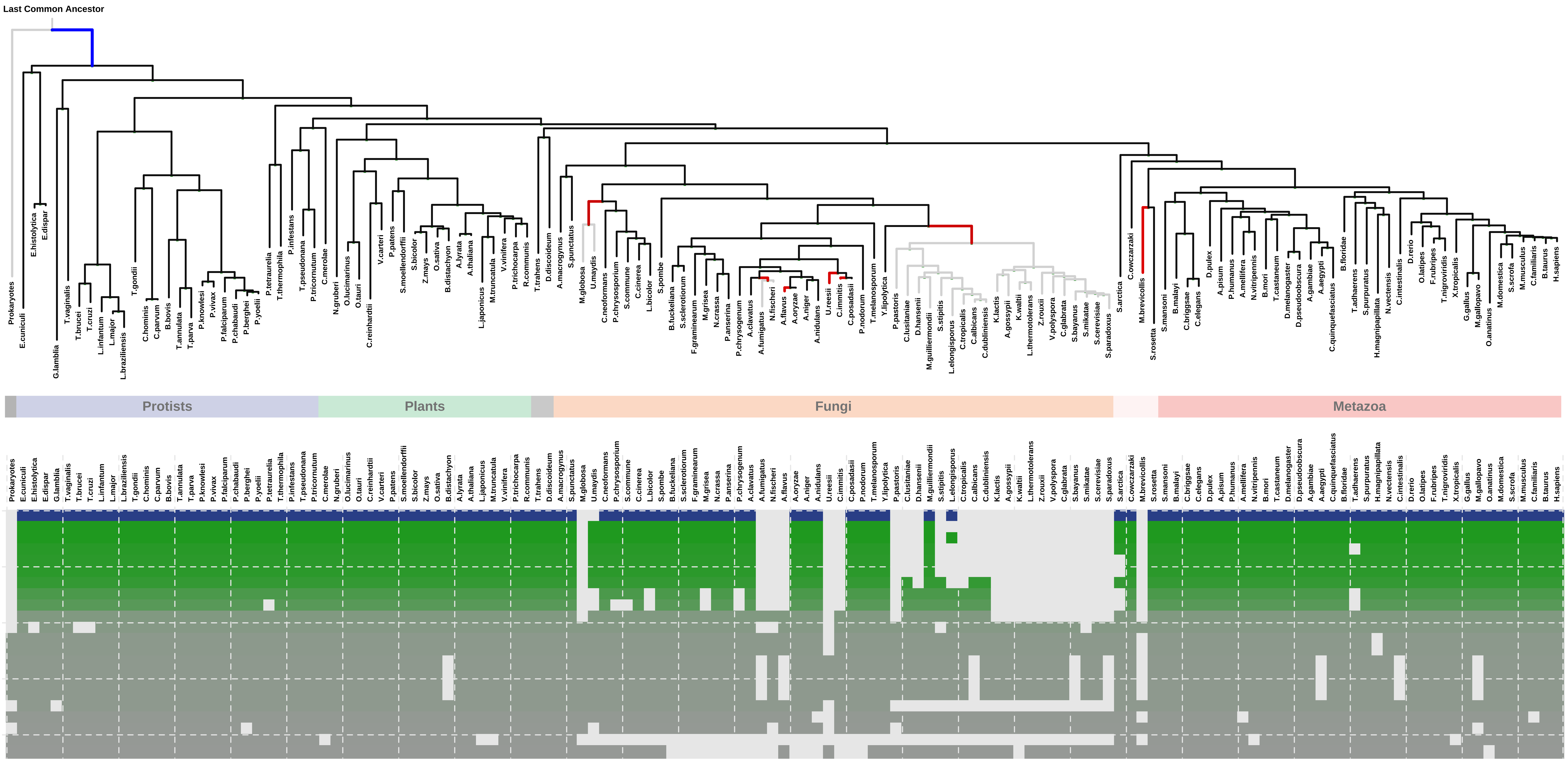

PG	Protein	LLR	Notes
A	AT4G31980		
A	AT5G47310	52.3	
A	AT5G25170	52.3	
A	AT1G47740	48.3	
A	AT2G25190	47.0	
A	AT1G80690	47.0	
A	AT4G17486	43.1	
A	AT4G25680	33.3	
A	AT4G25660	27.6	
A	AT3G07090	10.2	
	HUB2	8.1	
B	NRPB6A	5.6	
B	NRPB6B	5.6	
C	AT2G45710	4.9	
C	AT3G61111	4.9	
C	RS27A	4.9	
C	AT5G47930	4.9	
	AT4G27890	4.5	
	emb2191	2.6	
	AT5G14520	2.0	
	AT1G20410	1.7	
	AT3G63510	1.2	
