# Supplementary material for: Insight into the Characterization of Two Female Suppressor Gene Families: SOFF and SyGI in Plants
Source: Genes (Basel). 2025 Feb 26;16(3):280. doi: 10.3390/genes16030280 (PMC11941796; doi:10.3390/genes16030280)
Supplement: Supplementary file 1 [file genes-16-00280-s001.zip › File S2.pdf]

Output results of CLIME (CLustering by Inferred Models of Evolution)

Dataset:

Num of genes in input gene set: 2
Total number of genes: 27361
Prediction LLR threshold: 0

The CLIME PDF output two sections:

1) Overview of Evolutionarily Conserved Modules (ECMs)

- Top panel shows the predefined species tree.
- Bottom panel shows the partition of input genes into Evolutionary Conserved Modules (ECMs), ordered by ECM strength (shown at right), and separated by horizontal lines.
- Each row show one gene, where the phylogenetic profile indicates presence (blue) or absence (gray) of homologs in each species (column).
- Gene symbols are shown at left. Gray color indicates that the gene is a paralog to a higher scoring gene within the same ECM (based on BLASTP E < 1e-3).

2) Details of each ECM and its expansion ECM+

- Top panel shows the inferred evolutionary history on the predefined species tree. Branch color shows the gain event (blue) and loss events (red color, with brighter color indicating higher confidence in loss). Branches before the gain or after a loss are shown in gray.
- Bottom panel shows the input genes that are within the ECM (blue/white rows) as well as all genes in the expanded ECM+ (green/gray rows). The ECM+ includes genes likely to have arisen under the inferred model of evolution relative to a background model, and scored using a log likelihood ratio (LLR).
- PG indicates "paralog group" and are labeled alphabetically (i.e., A, B). The first gene within each paralog group is shown in black color. All other genes sharing sequence similarity (BLAST E < 1e-3) are assigned to the same PG label and displayed in gray.

Overview of Evolutionarily Conserved Modules (ECMs)

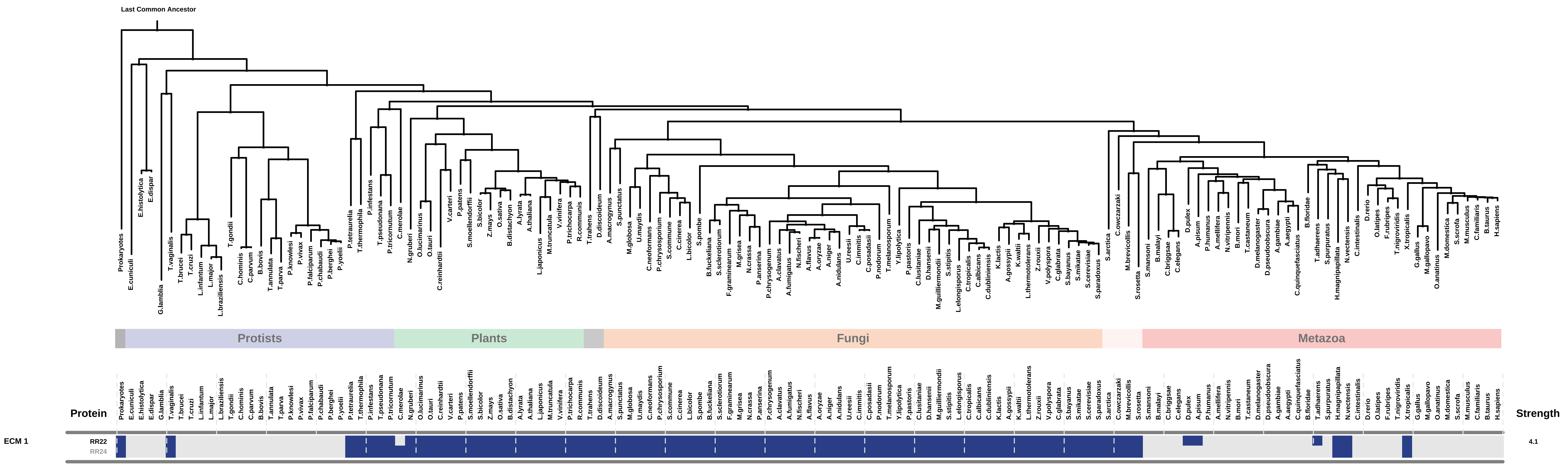

ECM 1, Page 1

Num of ECM Genes: 2. Num of Predicted Genes: 52. ECM Strength: 4.1

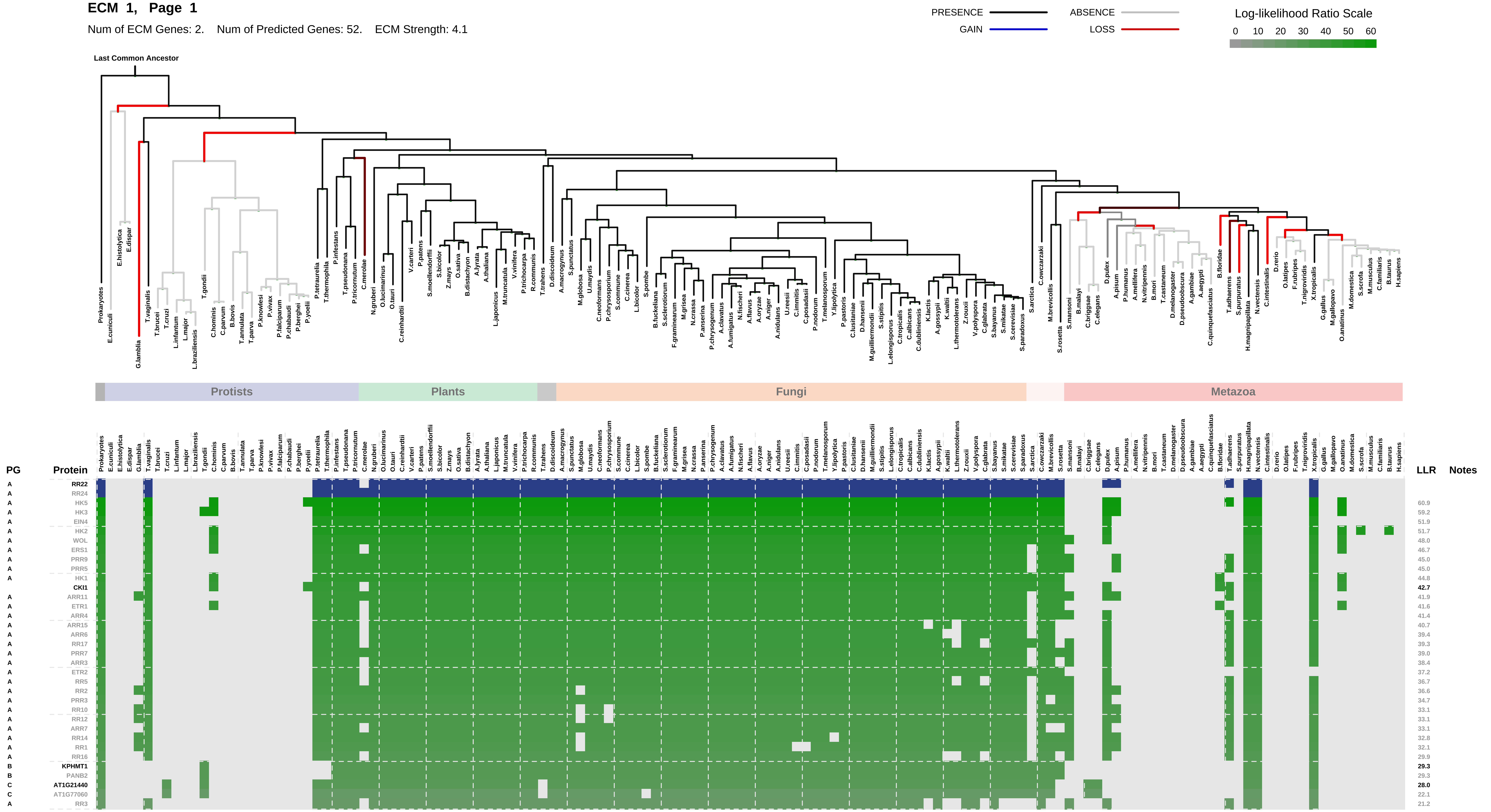

ECM 1, Page 2

Num of ECM Genes: 2. Num of Predicted Genes: 52. ECM Strength: 4.1

PRESENCE — ABSENCE
GAIN — LOSS

Log-likelihood Ratio Scale: 0 10 20 30 40 50 60

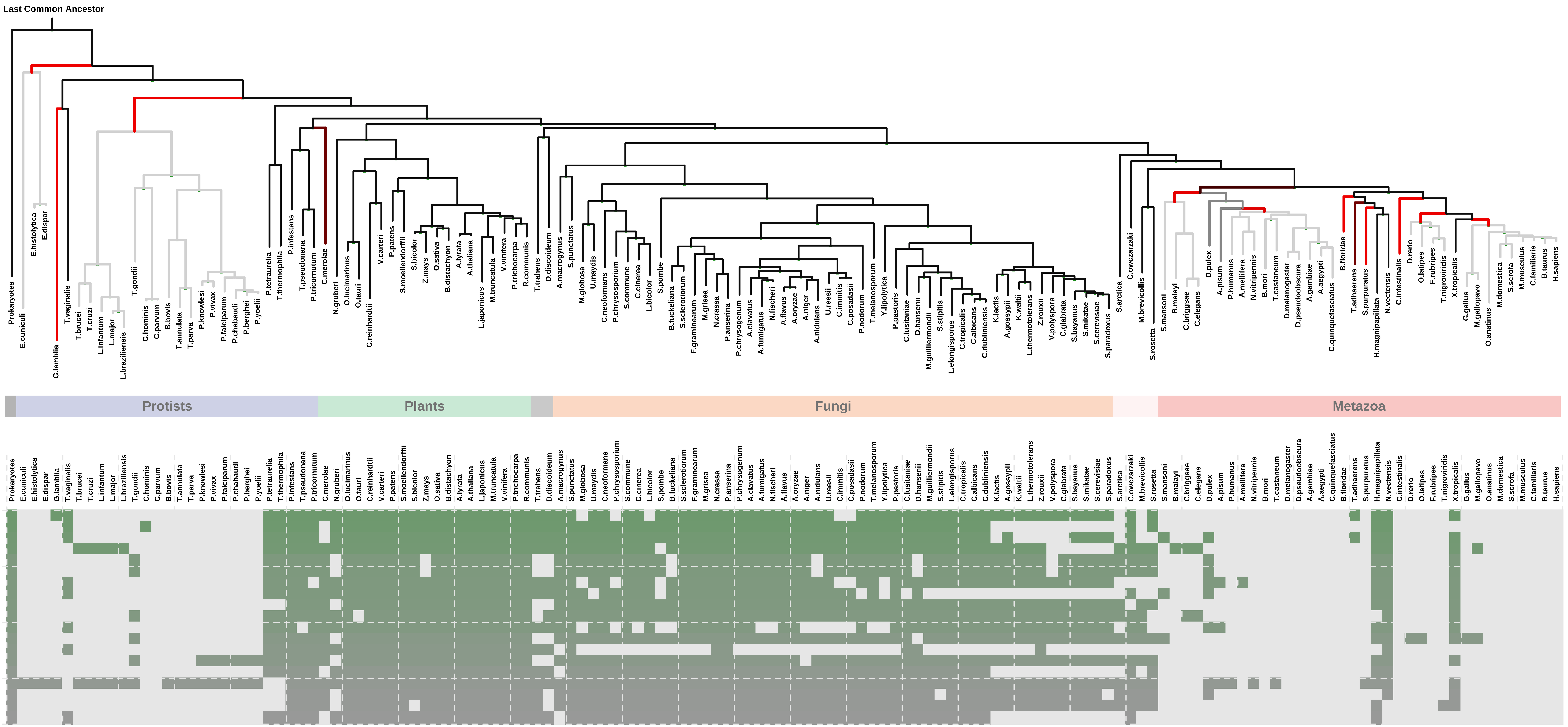

PG	Protein	LLR	Notes
A	RR18	18.4	
A	PHYA	17.9	
A	ARR9	16.3	
	FAD2	15.9	
D	AT2G45300	11.6	
D	AT1G48860	11.6	
A	RR20	11.2	
A	TOC1	11.1	
	AT3G23940	10.6	
C	ICL	9.8	
A	RR19	9.8	
	AT5G66120	7.1	
A	APRR4	6.6	
D	EMB1144	6.4	
A	PHYB	3.8	
	AT1G07200	2.4	
	HISN5B	1.4	
	HDH	1.3	
A	PHYC	0.4	
