# Supplementary figures and images for: Insight into the Characterization of Two Female Suppressor Gene Families: SOFF and SyGI in Plants
Source: Genes (Basel). 2025 Feb 26;16(3):280. doi: 10.3390/genes16030280 (PMC11941796; doi:10.3390/genes16030280)

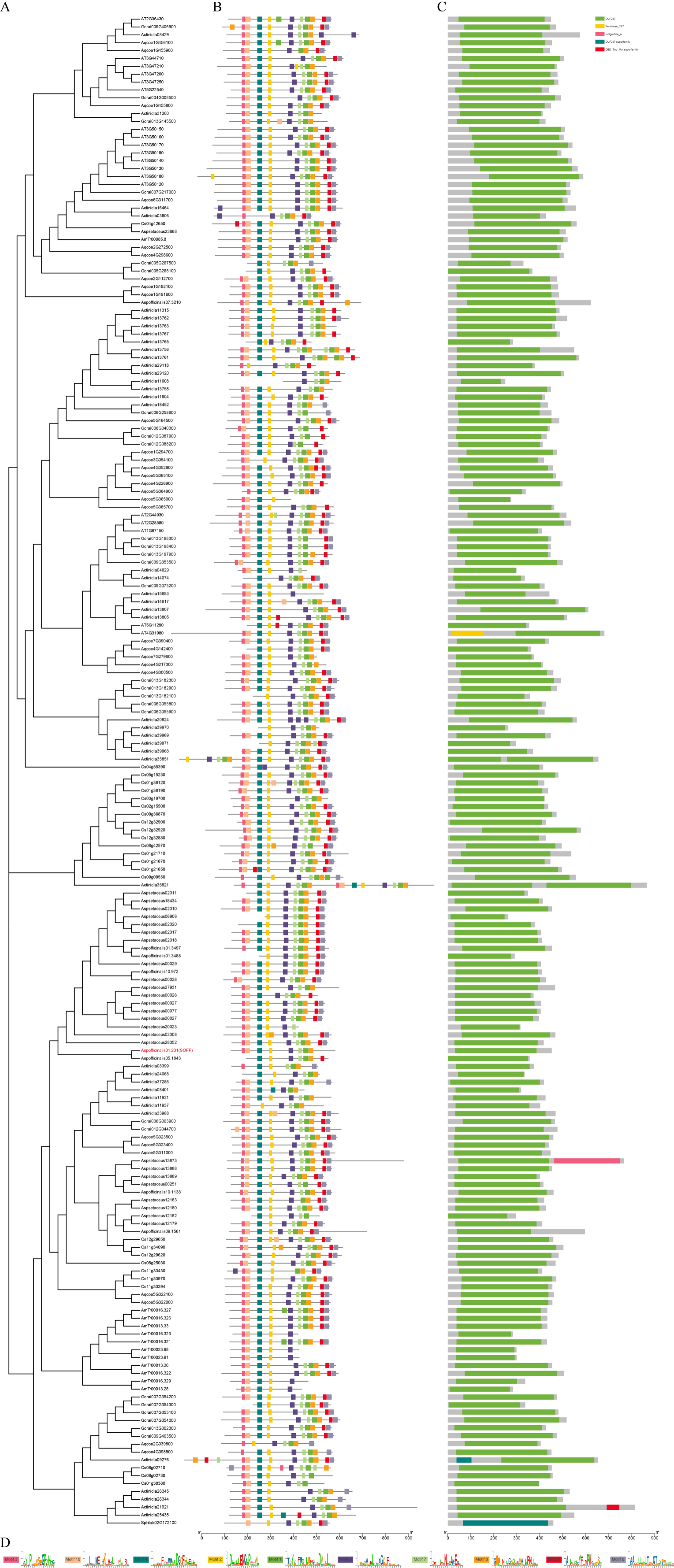

Supplement: Supplementary file 1 [file genes-16-00280-s001.zip › Figure S1.pdf]
